# A Comparison of Protein Kinases Inhibitor Screening Methods Using Both Enzymatic Activity and Binding Affinity Determination

**DOI:** 10.1371/journal.pone.0098800

**Published:** 2014-06-10

**Authors:** Amalie Frederikke Rudolf, Tine Skovgaard, Stefan Knapp, Lars Juhl Jensen, Jens Berthelsen

**Affiliations:** 1 Novo Nordisk Foundation Center for Protein Research, Faculty of Health Sciences, University of Copenhagen, Copenhagen, Denmark; 2 Costerton Biofilm Center, Institute for International Health, Immunology and Microbiology, Faculty of Health Sciences, University of Copenhagen, Copenhagen, Denmark; 3 Structural Genomics Consortium, Nuffield Department of Clinical Medicine, University of Oxford, Oxford, United Kingdom; Islamic Azad University-Mashhad Branch, Mashhad, Iran, Iran, Republic of Islamic

## Abstract

Binding assays are increasingly used as a screening method for protein kinase inhibitors; however, as yet only a weak correlation with enzymatic activity-based assays has been demonstrated. We show that the correlation between the two types of assays can be improved using more precise screening conditions. Furthermore a marked improvement in the correlation was found by using kinase constructs containing the catalytic domain in presence of additional domains or subunits.

## Introduction

Protein kinases constitute a large group of evolutionary and structurally related enzymes that regulate the function of other proteins by phosphorylation of a serine, threonine, or tyrosine residue in the target protein. Beside the catalytically active domain, many kinases contain additional domains that may regulate the kinase conformation and activity, as well as interactions with other proteins. Kinases are involved in mediation of signal transduction in virtually all cellular processes, including cell growth and differentiation [1]. Their involvement in regulation of such essential processes makes them attractive therapeutic targets, and a great number of high throughput kinase screens have been performed between industry and academia ([2]–[9]). Currently, 15 small-molecular kinase inhibitors have been approved for clinical use [10]–[12].

High-throughput screening platforms have proved beneficial for protein kinase drug discovery and development, as large compound libraries can be screened with ease and compound potency and selectivity across a subset of the kinome can be determined [13], [14]. Screening of kinases is frequently performed using the catalytic domain only, even though this has potential drawbacks as the presences of additional domains or subunits may affect the function of the kinase [15]. Traditionally, enzymatic assays are the preferred choice for screening. Such assays can, however, both be very expensive and technically challenging to run in high throughput mode, and alternative screening platforms that measure inhibitor binding independently of enzyme activity have gained popularity both in high-throughput and selectivity screening due to their economic and technical convenience [14], [16]. Inhibitor binding based assays are accepted as alternative screening platforms to enzymatic assays [4]–[6], [17].

The use of binding assays poses the question: to which extent does binding of an inhibitor imply inhibition of the catalytic activity? Several studies examining this have found a statistical significant but weak correlation between binding data and enzymatic activity data from published high-throughput screens of kinase domains [2], [5], [18], [19]. One likely reason for this weak correlation is, that the kinases used to perform the two types of assays were produced using different expression constructs, expression systems, and purification procedures [2], [5], [18], [19]. The convenience of bacterial expressions systems makes them attractive for producing kinases for binding assays. However, the risk is that inhibitor binding may be measured on an inactive form of the kinase and thus not be comparable to activity-based screens that use active kinases. To obtain catalytically active kinases, higher eukaryote expression systems are frequently used, which has the drawback that other kinases or phosphatases may be present as contaminants and influence activity measures, and that the produced proteins are often even more heterogeneous with respect to the posttranslational modifications that they harbor, as compared to recombinant proteins from bacterial expression systems.

To eliminate the influence of differences in expression constructs, expression systems, purification procedures and assay conditions, we expressed and purified a diverse selection of 14 protein kinases from *Escherichia coli*, and tested them against a panel of 240 well-characterized known kinases inhibitors employing both binding and enzymatic activity assays. For all 14 kinases, we expressed and purified the catalytic domain separately, and for 8, we also made a longer version containing one or more additional domains adding up to a total of 23 protein enzymes and variants, that were screened against a compound library of 244 protein kinase inhibitors. We show that a better correlation between activity- and binding-based screening data can be achieved by including extra domains in the constructs, by determining activity IC_50_ values for inhibitors (as opposed to determining inhibition percentage at a fixed inhibitor concentration), and by eliminating various error sources and inconsistencies between the screens.

## Results and Discussion

To obtain a homogeneous set of protein kinases to compare inhibitor binding and enzymatic inhibition, we set out to express and purify a diverse selection of 14 protein kinases from *Escherichia coli* using standardized procedures. For all of them, we expressed and purified the catalytic domain separately, and for 8, we also made a longer version containing one or more additional domains adding up to a total of 23 protein enzymes and variants. The protein kinases were all more than 85% pure. Of the 23 kinases, 6 did not show enzymatic activity, and we excluded these from the analysis. Two other kinases were excluded because the activity was too low to reliably determine IC_50_ values. The remaining 15 kinases (Table 1) were screened against a compound library of 244 well-characterized protein kinase inhibitors (listed in Table S1), which were selected to target all branches of the kinome. The activity and DSF screens were performed with the same purification batch of protein kinases in order to eliminate any potential variations owing to differences in expression and purification.

**Table 1:** Protein kinase constructs and properties.

Name (U-ID)	Domains	Uniprot seq	ATP Km(µM)	Assay ATP(µM)	Assay enzyme(µg/ml)	Assay substrate sequence
AKT3 (Q9Y243)	CD	122–479	564±36	1100	0.13	5F-GRPRTSSFAEG-CONH2
CDK2 (P24941)	C	1–299				
CCNA2 (P20248)	Cyclin A	75–432	238±12	480	5.0	5F-QSPKKG-CONH2
CCNE1 (P24864)	Cyclin E	43–381	145±4	480	8.1	5F-QSPKKG-CONH2
CHK2 (O96017)	CD	195–521	130±13	260	0.060	5F-KKLRRTLSVA-COOH
DMPK (Q09013)	CD	6–450	470±69	1000	9.2	5F-AKRRRLSSLRA-COOH
FES (P07332)	CD	423–694	49±11	100	0.005	5F-EFPIYDFLPAKKK-CONH2
FES (P07332)	CD+SH2	447–822	53±13	100	0.038	5F-EFPIYDFLPAKKK-CONH2
PAK4 (O96013)	CD	288–591	11±1	25	0.0015	5F-RRRLSFAEPG-CONH2
PAK4 (O96013)	CD+PBD	1–591	11±1	25	0.0017	5F-RRRLSFAEPG-CONH2
PAK7 (Q9P286)	CD	425–719	5±0.4	10	0.0015	5F-RRRLSFAEPG-CONH2
SRC (P12931)	CD	262–523	134±16	460	0.011	5F-EEPLYWSFPAKKK-CONH2
SRC (P12931)	CD+SH2/3	1–525	226±33	460	0.012	5F-EEPLYWSFPAKKK-CONH2
STK3 (Q13188)	CD	6–312	140±12	280	1.8	5F-KKSRGDYMTMQIG-CONH2
STK3 (Q13188)	CD+SARAH	6–490	66±2	280	1.0	5F-KKSRGDYMTMQIG-CONH2

Protein kinase constructs and properties. Overview of the constructsused in this study, indicating protein name and Uniprot-ID (U-ID), protein domain contents and the corresponding amino acids of the relative Uniprot sequences (FL: full length; CD: catalytic domain). Also indicated is K_m_ for ATP that we determined for each construct, the concentration of ATP present in the assay for each kinase, the amount of enzyme used and the assay substrate peptide sequence (5F: 5-FAM or 5- Fluorescein AMidite tag).

For the binding assay we used the well-established Differential Scanning Flourimetry (DSF) [20], [21], in which the thermal unfolding of a protein is monitored using a fluorescent dye. The dye is highly fluorescent in a nonpolar environment and quenched in aqueous solution, hereby making it possible to monitor the unfolding of a protein during heating. Binding of a compound to the native protein leads to stabilization of the protein, which is observed as shift in the melting temperature (T_m_) of the protein [20]. We performed DSF screening of the protein kinases in duplicate and repeated 3 times, which gave consistent data with an average standard deviation between replicates of 0.4 degrees (Table S2). Of the 15 kinase constructs screened, only the PAK2 catalytic domain did not work. DSF is generally not well suited for protein complexes, but it was nonetheless possible to screen CDK2 in complex with either cyclin A or E. In the DSF screen, the compound SB 218078 was observed to interfere with the fluorescence from the dye, likely due to intrinsic fluorescence. Another compound NH125 only produced negative Tm shifts indicating a preferred binding to the unfolded state of the protein.

We assessed the protein kinase enzymatic activity and measured the IC_50_ (Table 1 and S3) of each compound using mobility shift assay, which is based on Caliper’s microfluidics capillary electrophoresis. The kinase-mediated transfer of a phosphate group from ATP to a short peptide causes a change in the net charge of the peptide. The charge difference between the phosphorylated product and non-phosphorylated peptide substrate enables separation of the two in an electric field. Utilizing fluorescent-labeled peptides, real-time detection and quantification of both substrate and product is possible, and the reaction turnover can be determined [22]. The sensitivity of the mobility shift assay is comparable to that of radiometric assays [23]. A concern with activity-based screens is that most inhibitors compete with ATP for binding, for which reason the results depend on the ATP concentration [24]. Because the potency of ATP-competitive compounds is affected by the affinity of the kinase for ATP and the ATP concentration [24], we measured the K_m_ for ATP for each kinase construct, and used an ATP concentration of twice to four times the K_m_ for each individual kinase assay (Table 1). This ensures comparability of compound potency between the kinases. We performed initial primary screens at 10 µM compound concentration and based on this we selected the 32 compounds that gave the highest inhibition rate for subsequent IC_50_ determination. In this screen, a smaller subset of the compounds proved to be potent but not very selective, which has led them to be selected for IC_50_ determination for the majority of the protein kinases in the study (Table S3).

**Figure 1:**
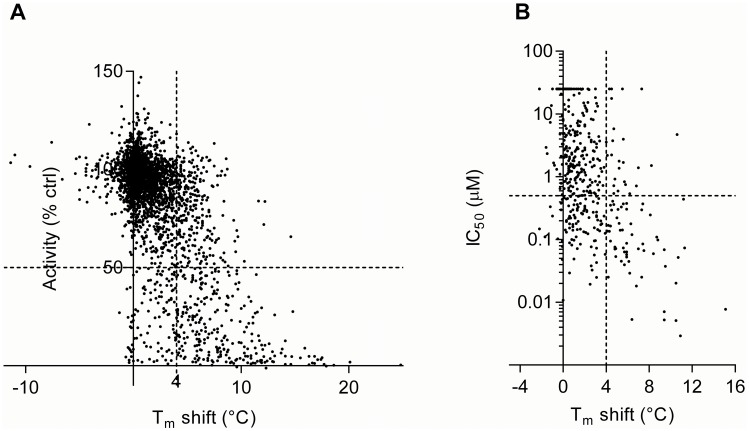
Binding and activity-based screening data comparison based on kinase-inhibitor pairs. **A**, Recreation of a scatter plot from Anastassiadis *et al*. [2] with comparison of their data, % activity of control (Ctrl) at 0.5 µM compound with T_m_ shift data obtained from Fedorov *et al.* [5]. (**B**) Scatter plot comparison of our IC_50_ and T_m_ shift data. The dashed horizontal line corresponds in **A** to 50% activity at 0.5 µM compound and in **B** to an IC_50_ of 0.5 µM. The dashed vertical line in the plots of 4°C Tm shift corresponds to a binding affinity <1 µM. A significant correlation of binding and inhibition is observed for both analyses (P<0.001).

Finally, we compared T_m_ shifts and activity data to see to which extent binding of a compound implies inhibition. For this purpose we set a threshold of 4°C for the T_m_ shifts, which usually corresponds to a binding affinity of <1 µM [5]. As a baseline, we include an earlier analysis by Anastassiadis *et al*., who compared activity measurements done at a fixed compound concentration at 0.5 µM and ATP at 10 µM [2], to T_m_ shifts from Fedorov *et al.* [5] (Figure 1A). 49% of the compounds with a T_m_ shift above 4°C show <50% catalytic activity, which implies IC_50_<0.5 µM. By comparison, the data on all our kinase constructs together yields better correlation: 71% of the compounds with T_m_>4°C have IC_50_<0.5 µM (Figure 1B). This shows that the observed agreement between binding and inhibition can be improved by using proteins from the same purification for both assays, and, perhaps more importantly, determining comparative IC_50_ values for compounds, prior to comparison.

**Figure 2:**
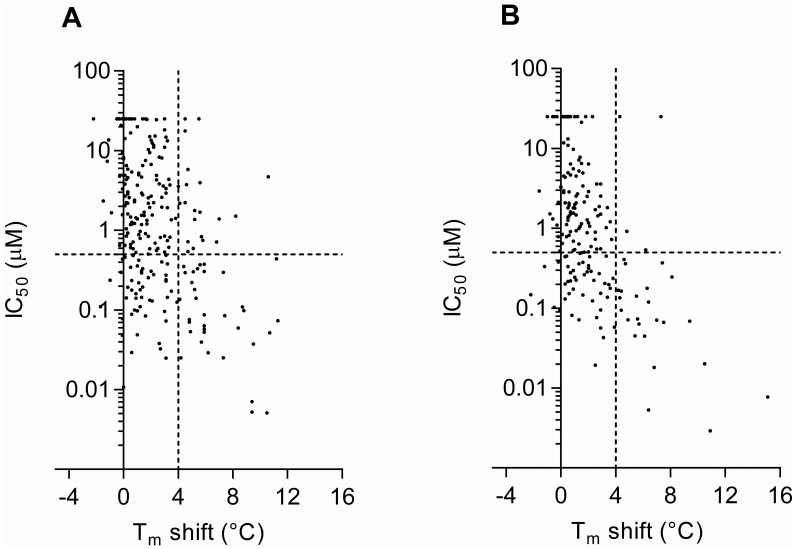
Binding and activity-based screening data comparison based on kinase-inhibitor pairs. Scatter plot comparison of our IC_50_ and T_m_ shift data divided into proteins with only the catalytic domains (**A**) and proteins with additional domains/subunits only (**B**). The dashed horizontal line corresponds to an IC_50_ of 0.5 µM. The dashed vertical line in the plots of 4°C Tm shift corresponds to a binding affinity <1 µM. A significant correlation of binding and inhibition is observed (P<0.001).

When we divide our kinase constructs into two groups of catalytic domain only in one group, and catalytic domain with additional domains in another, we find for the first group that the fraction of compounds with T_m_>4°C and IC_50_<0.5 µM is only 63% (Figure 2A). This fraction of compounds improves to 86%, when screening the catalytic domain together with additional domains or binding partners (Figure 2B). This correlation difference between the catalytic domain only group and the catalytic and other domains is statistically significant by a one-tailed Fisher’s exact test (P = 0.026). This implies that kinase activity is more consistent, and that screening results can be improved, by using a kinase construct containing addition domains than just the catalytic domain or by screening the catalytic subunit in the presence of subunits in a kinase complex. Another benefit of including additional domains or subunits in the screening is the possiblity of developing selective drugs by targeting other regions of the kinase than the highly conserved catalytic domain.

In summary, based on a large set of different proteins and inhibitors, we have shown that there exist a good correlation between measurements of inhibitor binding and enzymatic activity inhibition. This indicates that much of the disagreements between binding- and activity-based assays present in the public domain can be explained by the use of different constructs, expression systems, purification protocols and assay conditions, all of which lead to a poor comparability between different published inhibitor datasets. Another important improvement over other large screening sets is that we measure the Km for ATP of each kinase and use a concentration of ATP near to the Km for each individual kinase assay, and then measure precise IC_50_ values. This gives a much more accurate inhibition value as compared to utilizing the same fixed ATP concentration for all kinases, and measuring percentage inhibition at a fixed compound concentration.

We also find that that there is a marked improvement in comparability between binding and activity when working with proteins that contain additional domains or subunit, besides the catalytic domain. Regions flanking the kinase catalytic domains have often been implicated in the regulation of kinase activity. These N or C-terminal regulatory motifs either stabilize the active state or a particular inactive state of the kinase. This suggests that binding of inhibitors that recognize the active state (type I compounds) or the DFG out-state (type II compounds) is strongly influenced by regulatory motifs outside the catalytic domain. For instance tyrosine kinases of the Tec family, Csk, Abl or Fes/Fer require the N-terminal SH2 domain for activity [25], [26]. Some of the molecular mechanisms of SH2 kinase domain interaction have been elucidated by crystal structures. A shared mechanism in these diverse cytoplasmic tyrosine kinases is the stabilization of the active kinase state by the SH2 domain increasing the binding of type I inhibitors.

By using the improvements discussed above, the fraction of binding compounds that also show enzymatic inhibition increased from the 49% reported in a previous study [2] to 86%. This shows that binding-based assays can be a valid alternative and a good proxy for activity-based assays, especially if not performed on the kinase domain in isolation.

## Materials and Methods

### Protein Expression and Purification

The proteins used in this study (Table 1) were expressed in *Escherichia coli* using the expression vector pNIC28-Bsa4. The cells were lysed using a high-pressure homogenizer and cleared by centrifugation. The lysates were purified by immobilised metal ion chromatography (IMAC) followed by size exclusion chromatography (SEC). If purity was <85% additional purification was done either by His-tag cleavage followed by rebinding to IMAC or by ion exchange chromatography (IEX). The purified recombinant proteins were >85% pure as judged by SDS page, and protein identity was confirmed by DNA sequencing and mass spectrometry.

### Compounds

The 244 compounds are from Calbiochem Inhibitor Select 96-well protein kinase inhibitor Library I, II and III (Merck Millipore) (Table S1). The compounds are validated protein kinase inhibitors with the far majority of them targeting the ATP binding pocket. All compound were stored as a 1 mM stock in DMSO.

### Differential Scanning Fluorimetry

A LightCycler 480 Real-Time PCR System (Roche Applied Science) was used for the thermal denaturation experiments. The reactions were carried out in a 384 well plate in 5 µl reaction volume. A protein concentration of 2 µM and an inhibitor concentration of 10 µM were used. The sample buffer consisted of 100 mM HEPES (pH 7.5), 150 mM NaCl, 5 mM MgCl_2_ and 1∶2000 dilution of SyproOrange (Invitrogen). Temperature was increased stepwise 3.4°C/min from 20°C to 95°C. T_m_ shifts were calculated by LightCycler Tm Calling analysis software module.

### Mobility Shift Assay

Caliper EZ Reader II mobility shift assay was utilized for the activity-based screens. This assay is based on the difference in capillary electrophoresis mobility of a fluorescent-tagged peptide as a result of a phosphorylation by the studied kinase, hereby enabling direct detection of substrate to product conversion.

Reactions were started by the addition of 5 µl enzyme solution in assay buffer (100 mM HEPES (pH 7.5), 5 mM MgCl_2_, 0.05% CHAPS, 0.5 mM DTT and 0.1 mg/ml IgG) followed by 5 µl substrate mix containing ATP and peptide substrate in assay buffer. The peptide concentration was 1.5 µM. Concentrations of enzyme and ATP were adjusted to the specific requirements of the individual enzymes. ATP concentration was set to be within 2–4 fold of the K_m,ATP_ values and the enzyme concentration was set in order to give approximately 15% substrate conversion to ensure initial velocity measurements.

After incubation for 60 min at room temperature the kinase reaction was stopped by the addition of 5 µl EDTA to a final concentration of 5 mM. The stopped reaction was analyzed on a LabChip EZ Reader II (Caliper Life Science).

K_m_ values were determined at the assay conditions described above using 11 different ATP concentrations, ranging from 2048 µM to 2 µM in two-fold dilution steps and a no-ATP control. All ATP concentrations and the control are measured in duplicates. K_m_ values were determined by fitting the activity data to the Michaelis-Menten, Allosteric sigmoidal or Substrate inhibition functions using the built in equations in the Prism 5.0 software. K_m_ from the best fit was selected and an ATP concentration corresponding to 2–4 fold of the K_m_ was used for primary screening.

In the primary screen compounds were screened in duplicates at 10 µM. For the IC_50_ experiments the compounds were tested in duplicates from 0.1 nM to 25 µM in nine four-fold dilution steps. IC_50_ was determined once for each compound-kinase combination. Compounds were dissolved in DMSO resulting in 2.5% final DMSO concentration in all assays. Each assay plate contained compound dilutions for 16 compounds (C), 32 positive controls containing enzyme but no compound (P), and 32 negative controls containing no enzyme and no compound (N). For estimation of IC_50_, the % substrate conversion values are transformed to % relative activity by applying the following equation:
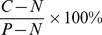



IC_50_ values were then calculated from dose-response curves using XLfit (curve fitting software for Excel).

### Statistical Analysis

Linear regression analyses were performed in GraphPad Prism 5.

Correlation between DSF and IC_50_ data was calculated as
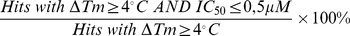



The significance of difference between protein construct groups were calculated using an one-tailed Fisher’s exact test (GraphPad Prism 5).

## Supporting Information

Table S1 **List of Kinase Inhibitor Compounds used in this study, displayed with structure and PubChem reference number.**
(PDF)

Table S2 **Melting temperature (T_m_) shift data, expressed in degrees Celcius, for each compound versus kinase.** Average of 3 experiments, each in duplicate.
(PDF)

Table S3 **IC_50_ values, expressed in µM, measured for the 32 most potent compounds for each kinase.** Average of three experiments.
(PDF)
