# Supplementary material for: A Comparison of Protein Kinases Inhibitor Screening Methods Using Both Enzymatic Activity and Binding Affinity Determination
Source: PLoS One. 2014 Jun 10;9(6):e98800. doi: 10.1371/journal.pone.0098800 (PMC4051630; doi:10.1371/journal.pone.0098800)
Supplement: Table S1 — List of Kinase Inhibitor Compounds used in this study, displayed with structure and PubChem reference number. (PDF) [file pone.0098800.s001.pdf]

## Table S1

| Nr | Compound name | CAS | M.W. | Structure | PubChem Ref |
|---|---|---|---|---|---|
| 1 | AG 1024 | 65678-07-1 | 305.2 | 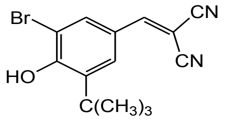 | 2044 |
| 2 | AGL 2043 | 226717-28-8 | 280.4 | 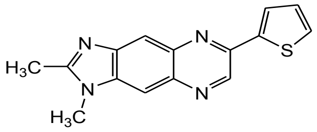 | 9817165 |
| 3 | Akt Inhibitor IV | 681281-88-9 | 614.6 | 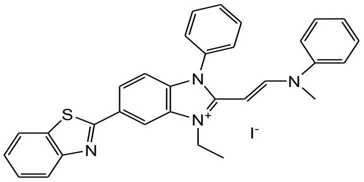 | 5719375 |
| 4 | Akt Inhibitor V, Triciribine | 35943-35-2 | 320.3 | 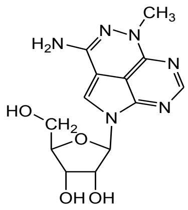 | 290486 |
| 5 | Akt Inhibitor VIII, Isozyme-Selective, Akti-1/2 | 612847-09-3 | 551.6 | 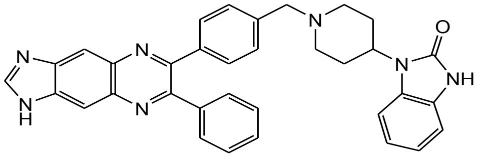 | 10196499 |
| 6 | Akt Inhibitor X | 925681-41-0 | 381.4 | 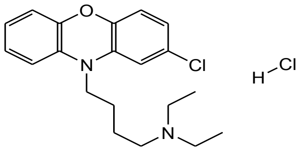 | 16760284 |
| 7 | PDK1/Akt/Flt Dual Pathway Inhibitor | 331253-86-2 and 329710-24-9 | 224.2 | 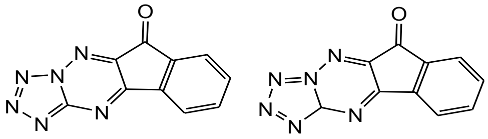 | 5113385 |
| 8 | Aurora Kinase Inhibitor II | 331770-21-9 | 400.4 | 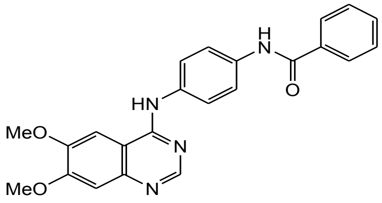 | 6610278 |

| | | | | | |
|---|---|---|---|---|---|
| 9 | Bcr-abl Inhibitor | 778270-11-4 | 374.3 | 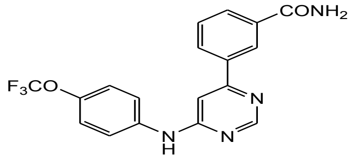 | 5311510 |
| 10 | Bisindolylmaleimide I | 133052-90-1 | 412.5 | 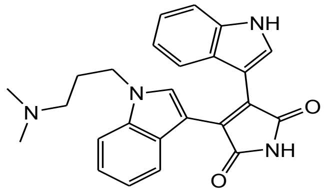 | 2396 |
| 11 | Bisindolylmaleimide IV | 119139-23-0 | 327.3 | 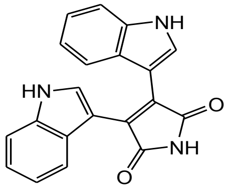 | 2399 |
| 12 | BPIQ-I | 174709-30-9 | 354.2 | 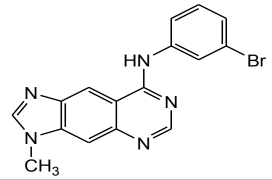 | 2427 |
| 13 | Chelerythrine Chloride | 3895-92-9 | 383.8 | 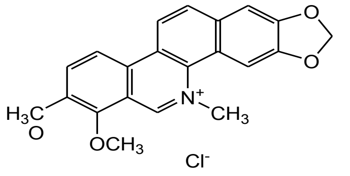 | 72311 |
| 14 | Compound 56 | 171745-13-4 | 388.8 | 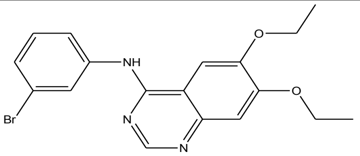 | 2857 |
| 15 | DNA-PK Inhibitor II | 154447-35-5 | 281.3 | 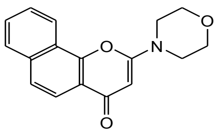 | 9860529 |
| 16 | DNA-PK Inhibitor III | 404009-40-1 | 221.3 | 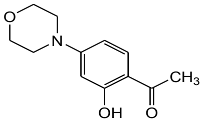 | 9859309 |
| 17 | PI-103 | 371935-74-9 | 348.4 | 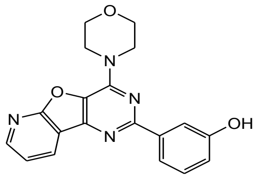 | 9884685 |

| | | | | | |
|---|---|---|---|---|---|
| 18 | Diacylglycerol Kinase Inhibitor II | 120166-69-0 | 489.6 | 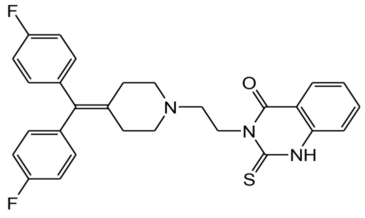 | 657356 |
| 19 | DMBI | 5812-07-7 | 264.3 | 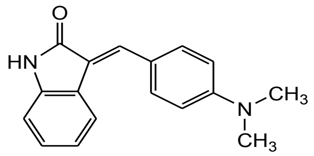 | 5353593 |
| 20 | EGFR/ErbB-2 Inhibitor | 179248-61-4 | 387.4 | 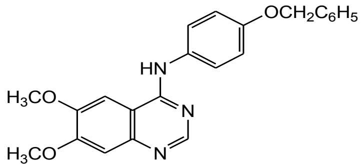 | 9843206 |
| 21 | EGFR Inhibitor | 879127-07-8 | 413.4 | 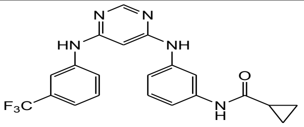 | 9549299 |
| 22 | EGFR/ErbB-2/ErbB-4 Inhibitor | 881001-19-0 | 355.8 | 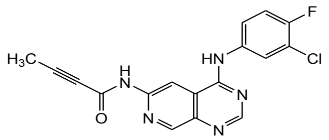 | 11566580 |
| 23 | Flt-3 Inhibitor | 301305-73-7 | 360.4 | 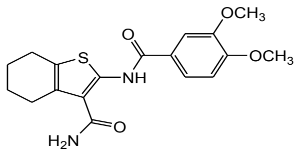 | 1048845 |
| 24 | Flt-3 Inhibitor II | 896138-40-2 | 292.3 | 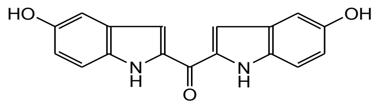 | 11601743 |
| 25 | cFMS Receptor Tyrosine Kinase Inhibitor | 870483-87-7 | 366.4 | 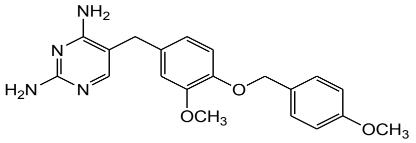 | 11617559 |
| 26 | Gö 6976 | 136194-77-9 | 378.4 | 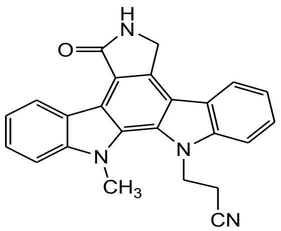 | 3501 |
| 27 | Gö 6983 | 133053-19-7 | 442.5 | 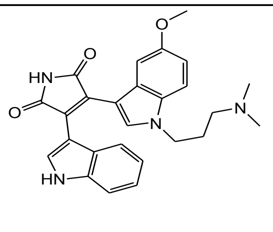 | 3499 |

| | | | | | |
|---|---|---|---|---|---|
| 28 | GTP-14564 | 34823-86-4 | 234.3 | 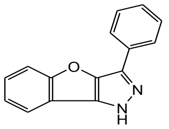 | 3385203 |
| 29 | Herbimycin A, Streptomyces sp. | 70563-58-5 | 574.7 | 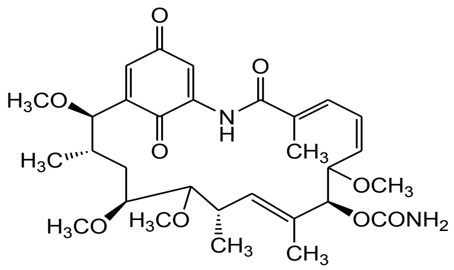 | 16760502 |
| 30 | Flt-3 Inhibitor III | 852045-46-6 | 365.5 | 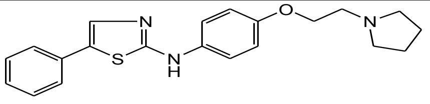 | 11772958 |
| 31 | IGF-1R Inhibitor II | 196868-63-0 | 341.8 | 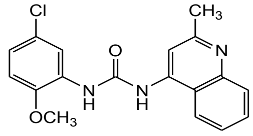 | 9549305 |
| 32 | IRAK-1/4 Inhibitor | 509093-47-4 | 395.4 | 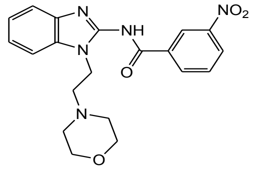 | 11983295 |
| 33 | JAK Inhibitor I | 457081-03-7 | 309.3 | 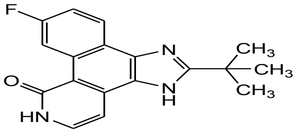 | 5494425 |
| 34 | JAK3 Inhibitor II | 211555-04-3 | 376.2 | 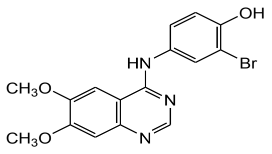 | 3795 |
| 35 | JAK3 Inhibitor IV | 58753-54-1 | 367.9 | 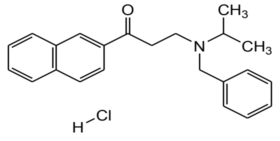 | 176406 |
| 36 | JAK3 Inhibitor VI | 856436-16-3 | 383.4 | 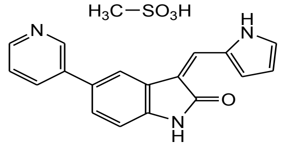 | 16760524 |

| | | | | | |
|---|---|---|---|---|---|
| 37 | Lck Inhibitor | 213743-31-8 | 370.5 | 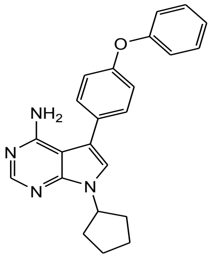 | 6603792 |
| 38 | LY 294002 | 154447-36-6 | 307.4 | 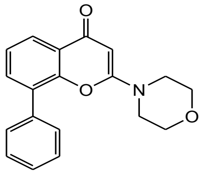 | 3973 |
| 39 | LY 303511 | 154447-38-8 | 306.4 | 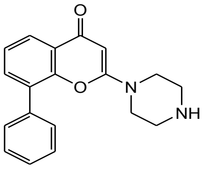 | 3971 |
| 40 | Met Kinase Inhibitor | 658084-23-2 | 568.1 | 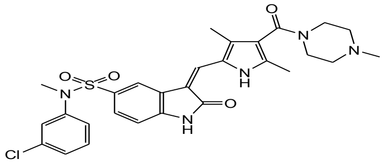 | 9549297 |
| 41 | PD 158780 | 171179-06-9 | 330.2 | 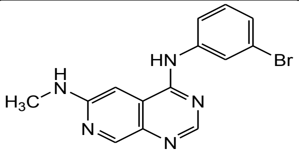 | 4707 |
| 42 | PD 174265 | 216163-53-0 | 371.2 | 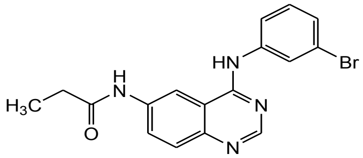 | 4709 |
| 43 | PDGF Receptor Tyrosine Kinase Inhibitor II | 249762-74-1 | 346.4 | 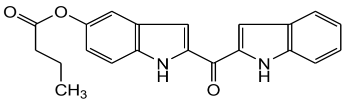 | 5330548 |
| 44 | PDGF Receptor Tyrosine Kinase Inhibitor III | 205254-94-0 | 485.5 | 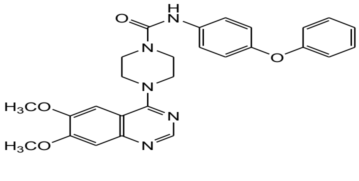 | 10907042 |

| | | | | | |
|---|---|---|---|---|---|
| 45 | PDGF Receptor Tyrosine Kinase Inhibitor IV | 627518-40-5 | 325.3 | 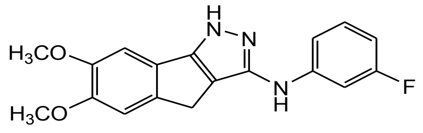 | 9797370 |
| 46 | PDGF RTK Inhibitor | 347155-76-4 | 527.6 | 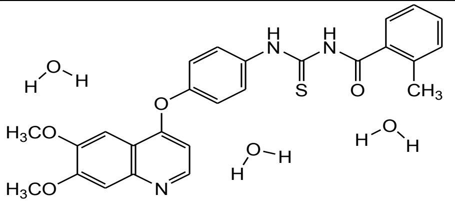 | 16760609 |
| 47 | PKR Inhibitor | 608512-97-6 | 268.3 | 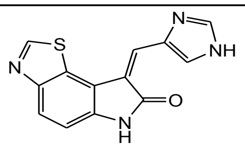 | 6490494 |
| 48 | PKR Inhibitor, Negative Control | 852547-30-9 | 340.6 | 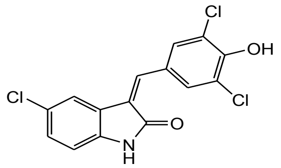 | 16760619 |
| 49 | PI 3-Kγ Inhibitor | 648450-29-7 | 257.3 | 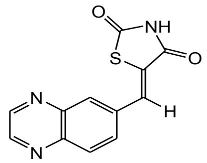 | 5289247 |
| 50 | PI 3-Kβ Inhibitor II | 648449-76-7 | 285.2 | 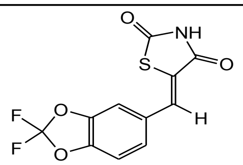 | 5287855 |
| 51 | PP3 | 5334-30-5 | 211.2 | 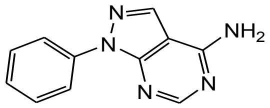 | 4879 |
| 52 | PP1 Analog II, 1NM-PP1 | 221244-14-0 | 331.4 | 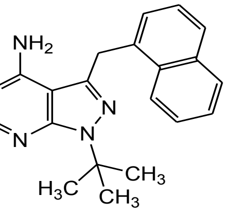 | 5154691 |
| 53 | PKCβII/EGFR Inhibitor | 145915-60-2 | 365.3 | 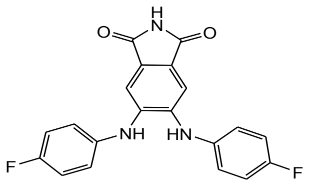 | 6711154 |

| | | | | | |
|---|---|---|---|---|---|
| 54 | PKCβ Inhibitor | 257879-35-9 | 411.5 | 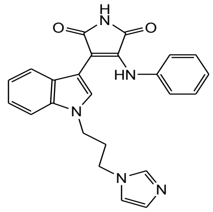 | 6419755 |
| 55 | Rapamycin | 53123-88-9 | 914.2 | 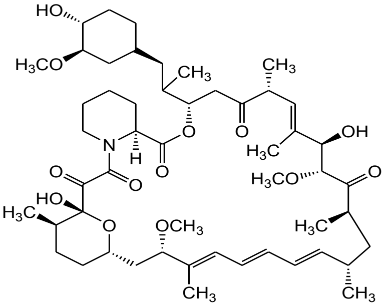 | 16760631 |
| 56 | Rho Kinase Inhibitor III, Rockout | 7272-84-6 | 194.2 | 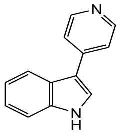 | 644354 |
| 57 | Rho Kinase Inhibitor IV | not registered | 467.4 | 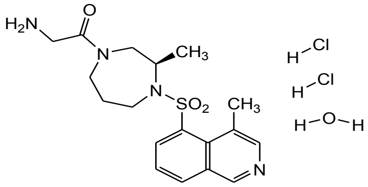 | 16760635 |
| 58 | Staurosporine, N-benzoyl- | 120685-11-2 | 570.6 | 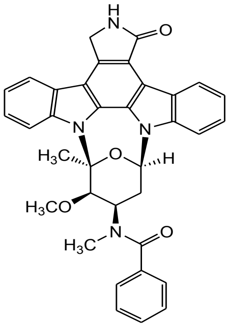 | 16760627 |
| 59 | Src Kinase Inhibitor I | 179248-59-0 | 373.4 | 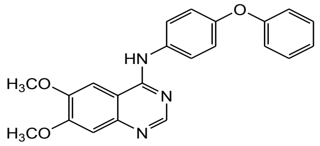 | 1474853 |
| 60 | SU11652 | 326914-10-7 | 414.9 | 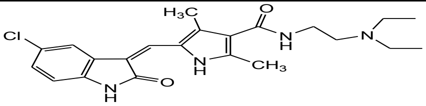 | 5329103 |
| 61 | Syk Inhibitor | 622387-85-3 | 353.4 | 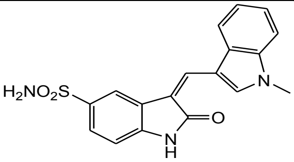 | 6419747 |
| 62 | Syk Inhibitor II | 227449-73-2 | 449.3 | 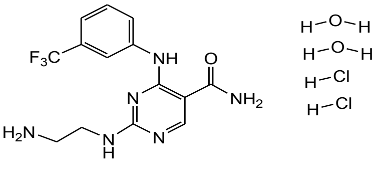 | 16760670 |

| | | | | | |
|---|---|---|---|---|---|
| 63 | Syk Inhibitor III | 1485-00-3 | 193.2 | 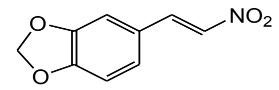 | 672296 |
| 64 | TGF-β RI Kinase Inhibitor | 396129-53-6 | 272.3 | 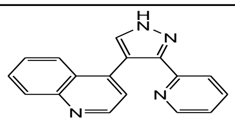 | 447966 |
| 65 | TGF-β RI Inhibitor III | 356559-13-2 | 371.9 | 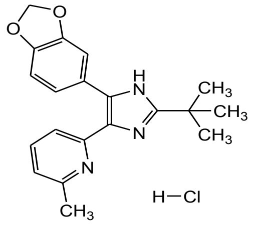 | 16079009 |
| 66 | AG 9 | 2826-26-8 | 184.2 | 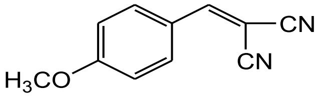 | 2063 |
| 67 | AG 490 | 133550-30-8 | 294.3 | 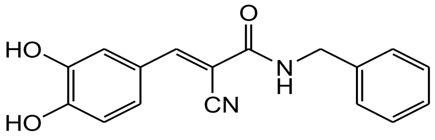 | 5328779 |
| 68 | AG 112 | 144978-82-5 | 236.2 | 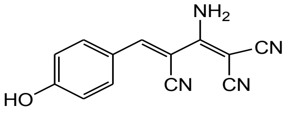 | 5328804 |
| 69 | AG 1295 | 71897-07-9 | 234.3 | 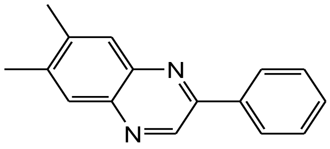 | 2048 |
| 70 | AG 1296 | 146535-11-7 | 266.3 | 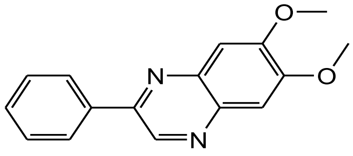 | 2049 |
| 71 | AG 1478 | 175178-82-2 | 315.8 | 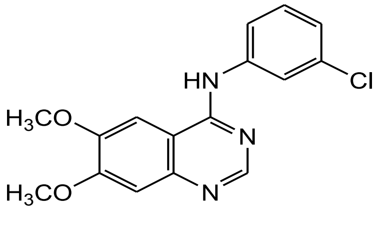 | 2051 |

| | | | | | |
|---|---|---|---|---|---|
| 72 | VEGF Receptor 2 Kinase Inhibitor I | 15966-93-5 | 310.4 | 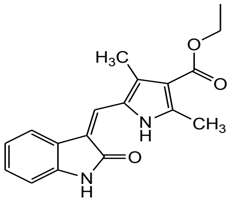 | 6419834 |
| 73 | VEGF Receptor Tyrosine Kinase Inhibitor II | 269390-69-4 | 337.8 | 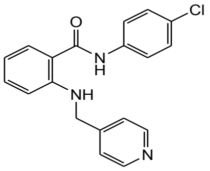 | 9797919 |
| 74 | VEGF Receptor Tyrosine Kinase Inhibitor III, KRN633 | 286370-15-8 | 416.9 | 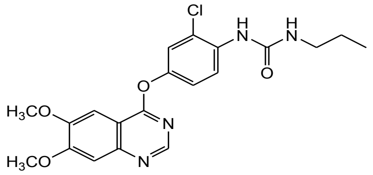 | 9549295 |
| 75 | VEGF Receptor 2 Kinase Inhibitor II | 288144-20-7 | 343.2 | 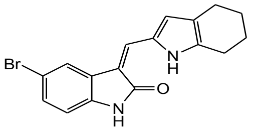 | 5329155 |
| 76 | VEGF Receptor 2 Kinase Inhibitor III | 204005-46-9 | 238.3 | 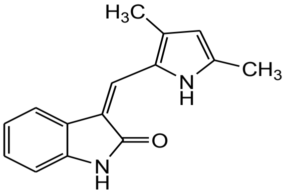 | 5329098 |
| 77 | VEGF Receptor 2 Kinase Inhibitor IV | 216661-57-3 | 307.4 | 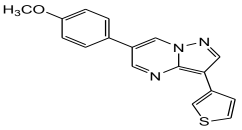 | 5329468 |
| 78 | DNA-PK Inhibitor V | 404009-46-7 | 283.3 | 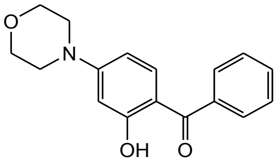 | 16760391 |
| 79 | Aurora Kinase Inhibitor III | 879127-16-9 | 413.4 | 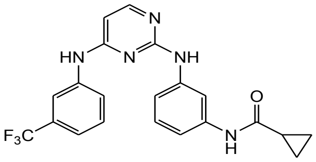 | 9549303 |

| | | | | | |
|---|---|---|---|---|---|
| 80 | Staurosporine, Streptomyces sp. | 62996-74-1 | 466.5 | 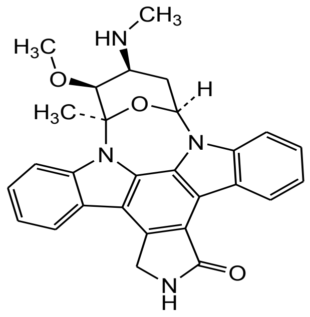 | 451705 |

| | | | | | |
|---|---|---|---|---|---|
| 81 | KN-62 | 127191-97-3 | 721.9 | 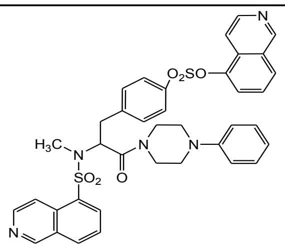 | 16760529 |
| 82 | ATM Kinase Inhibitor | 587871-26-9 | 395.5 | 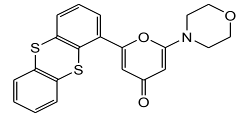 | 5278396 |
| 83 | ATM/ATR Kinase Inhibitor | 905973-89-9 | 555.8 | 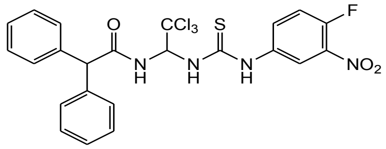 | 6605258 |
| 84 | Alsterpaullone | 237430-03-4 | 293.3 | 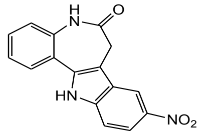 | 5005498 |
| 85 | Alsterpaullone, 2-Cyanoethyl | 852527-97-0 | 346.3 | 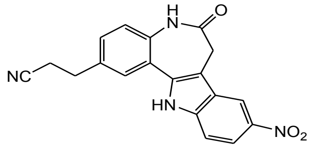 | 16760286 |
| 86 | Aloisine A, RP107 | 496864-16-5 | 267.3 | 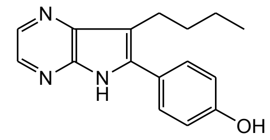 | 5326843 |
| 87 | Aloisine, RP106 | 496864-15-4 | 281.4 | 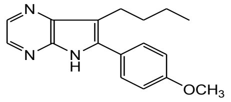 | 3641059 |
| 88 | Aminopurvalanol A | 220792-57-4 | 403.9 | 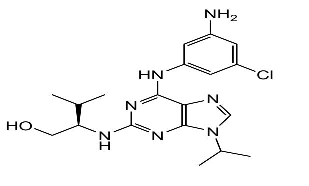 | 6604931 |
| 89 | AMPK Inhibitor, Compound C | 866405-64-3 | 399.5 | 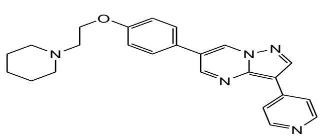 | 11524144 |

| | | | | | |
|---|---|---|---|---|---|
| 90 | Aurora Kinase Inhibitor III | 879127-16-9 | 413.4 | 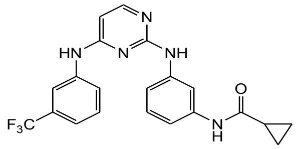 | 9549303 |
| 91 | Aurora Kinase/Cdk Inhibitor | 443797-96-4 | 435.4 | 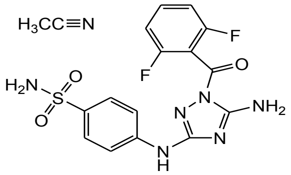 | 16760303 |
| 92 | Indirubin-3′-monoxime | 160807-49-8 | 277.3 | 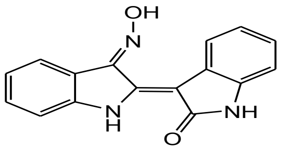 | 5326739 |
| 93 | BAY 11-7082 | 19542-67-7 | 207.2 | 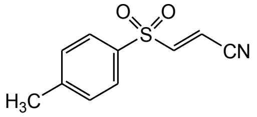 | 5353431 |
| 94 | Bohemine | 189232-42-6 | 340.4 | 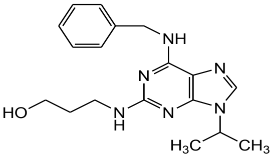 | 2422 |
| 95 | Cdk1 Inhibitor | 220749-41-7 | 294.7 | 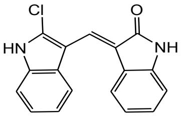 | 5472558 |
| 96 | Cdk1 Inhibitor, CGP74514A | 190654-01-4 | 385.9 | 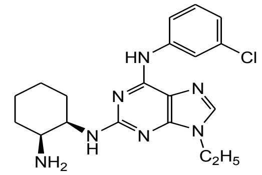 | 2794188 |
| 97 | Cdk1/2 Inhibitor III | 443798-55-8 | 425.4 | 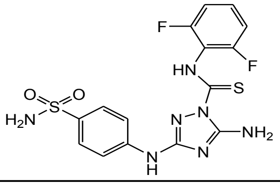 | 5330812 |
| 98 | Cdk1/5 Inhibitor | 40254-90-8 | 185.2 | 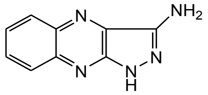 | 438981 |
| 99 | Casein Kinase I Inhibitor, D4476 | 301836-43-1 | 398.4 | 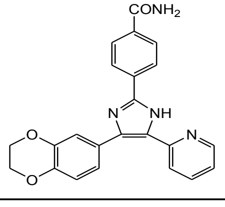 | 6419753 |

| | | | | | |
|---|---|---|---|---|---|
| 100 | Casein Kinase II Inhibitor III, TBCA | 934358-00-6 | 463.8 | 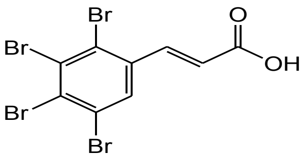 | 16760346 |
| 101 | Cdk4 Inhibitor | 546102-60-7 | 404.2 | 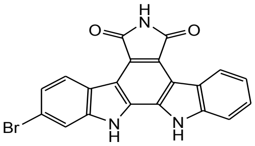 | 5330797 |
| 102 | Cdk4 Inhibitor II, NSC 625987 | 141992-47-4 | 271.3 | 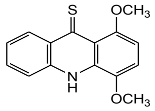 | 3004085 |
| 103 | Cdk4 Inhibitor III | 265312-55-8 | 284.3 | 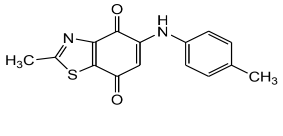 | 481747 |
| 104 | Cdc2-Like Kinase Inhibitor, TG003 | 300801-52-9 | 249.3 | 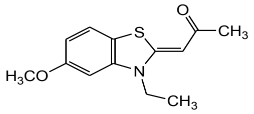 | 1893668 |
| 105 | Chk2 Inhibitor II | 516480-79-8 | 363.8 | 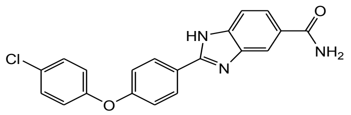 | 9969021 |
| 106 | Compound 52 | 212779-48-1 | 346.8 | 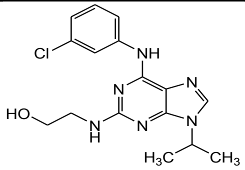 | 2856 |
| 107 | Cdk2 Inhibitor III | 199986-75-9 | 400.5 | 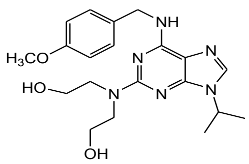 | 6918386 |
| 108 | Cdk2 Inhibitor IV, NU6140 | 444723-13-1 | 422.5 | 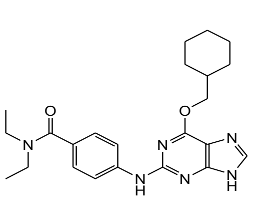 | 10202471 |
| 109 | Cdk/Crk Inhibitor | 784211-09-2 | 473.4 | 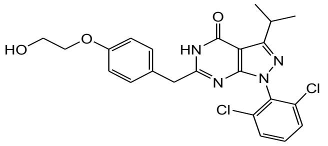 | 9549301 |

| | | | | | |
|---|---|---|---|---|---|
| 110 | ERK Inhibitor III | | 318.3 | 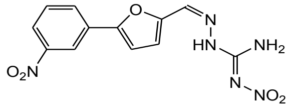 | 5339183 |
| 111 | ROCK Inhibitor, Y-27632 | 146986-50-7 | 338.3 | 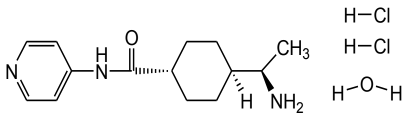 | 9797929 |
| 112 | ERK Inhibitor II, FR180204 | 865362-74-9 | 327.3 | 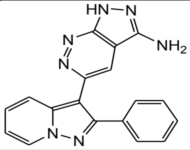 | 11493598 |
| 113 | ERK Inhibitor II, Negative control | | 328.3 | 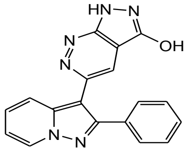 | 16760417 |
| 114 | Fascaplysin, Synthetic | | 306.8 | 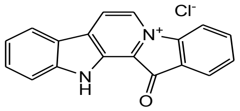 | 73292 |
| 115 | GSK-3b Inhibitor I | | 222.3 | 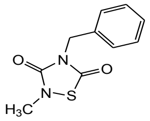 | 4124851 |
| 116 | GSK-3b Inhibitor II | 478482-75-6 | 395.2 | 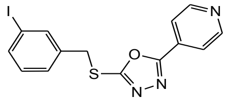 | 6539732 |
| 117 | GSK-3b Inhibitor VIII | 487021-52-3 | 308.3 | 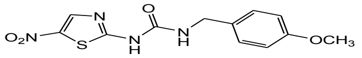 | 448014 |
| 118 | GSK-3 Inhibitor IX | 667463-62-9 | 356.2 | 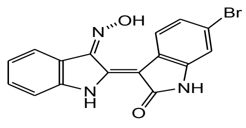 | 5287844 |
| 119 | GSK-3 Inhibitor X | | 398.2 | 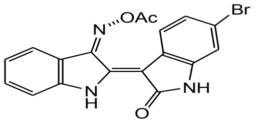 | 6538818 |
| 120 | GSK-3b Inhibitor XI | 626604-39-5 | 349.3 | 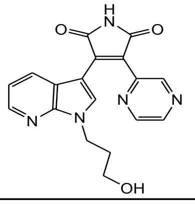 | 10020713 |

| | | | | | |
|---|---|---|---|---|---|
| 121 | SU6656 | 330161-87-0 | 371.5 | 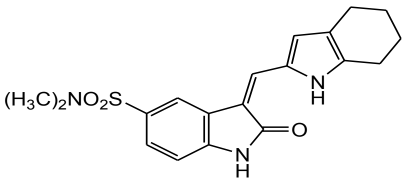 | 5312137 |
| 122 | GSK-3 Inhibitor XIII | 404828-08-6 | 301.4 | 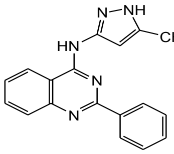 | 6419766 |
| 123 | Isogranulatimide | 244148-46-7 | 276.3 | 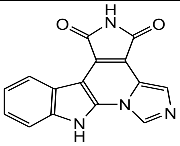 | 6419741 |
| 124 | IC261 | 186611-52-9 | 311.3 | 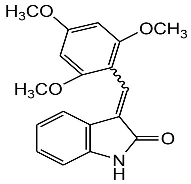 | 3674 |
| 125 | IKK-2 Inhibitor IV | 507475-17-4 | 279.3 | 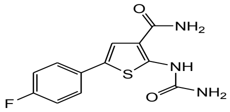 | 9903786 |
| 126 | Indirubin Derivative E804 | | 365.4 | 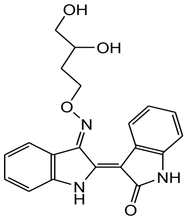 | 6419764 |
| 127 | JNK Inhibitor II | 129-56-6 | 220.2 | 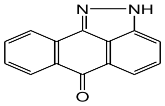 | 8515 |
| 128 | JNK Inhibitor, Negative Control | | 234.2 | 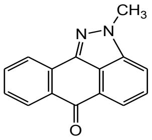 | 11665831 |
| 129 | JNK Inhibitor V | 345987-15-7 | 372.5 | 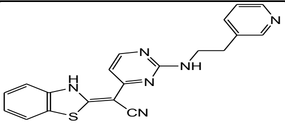 | 11422035 |
| 130 | JNK Inhibitor IX | 312917-14-9 | 350.4 | 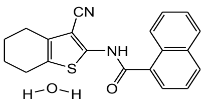 | 16760525 |
| 131 | MK2a Inhibitor | 41179-33-3 | 349.4 | 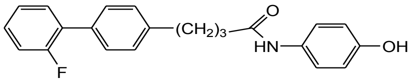 | 11382492 |

| | | | | | |
|---|---|---|---|---|---|
| 132 | JNK Inhibitor VIII | 894804-07-0 | 356.4 | 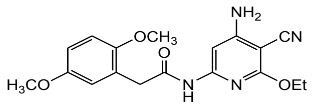 | 11624601 |
| 133 | K-252a, Nocardiopsis sp. | 97161-97-2 | 467.5 | 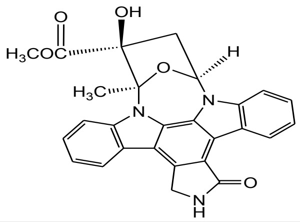 | 490561 |
| 134 | Kenpaullone | 142273-20-9 | 327.2 | 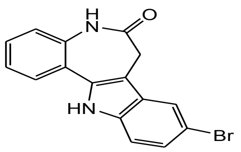 | 3820 |
| 135 | KN-93 | 139298-40-1 | 501.0 | 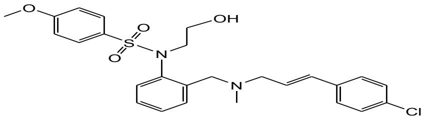 | 5312122 |
| 136 | MEK Inhibitor I | | 374.5 | 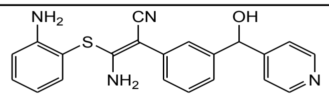 | 9951490 |
| 137 | MEK Inhibitor II | 623163-52-0 | 289.7 | 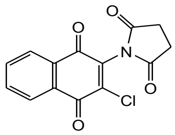 | 389898 |
| 138 | MEK1/2 Inhibitor | 305350-87-2 | 335.4 | 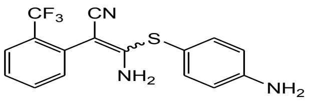 | 9549284 |
| 139 | MNK1 Inhibitor | 522629-08-9 | 244.2 | 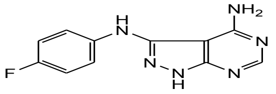 | 11644425 |
| 140 | NF-κB Activation Inhibitor | 545380-34-5 | 356.4 | 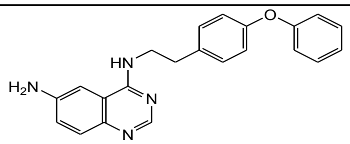 | 509554 |
| 141 | p38 MAP Kinase Inhibitor III | 581098-48-8 | 404.5 | 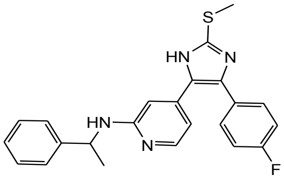 | 6419739 |

| | | | | | |
|---|---|---|---|---|---|
| 142 | p38 MAP Kinase Inhibitor | 219138-24-6 | 365.8 | 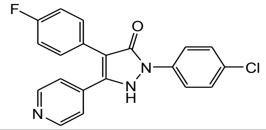 | 4665 |
| 143 | PD 98059 | 167869-21-8 | 267.3 | 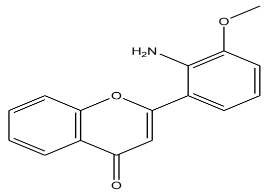 | 4713 |
| 144 | PD 169316 | 152121-53-4 | 360.3 | 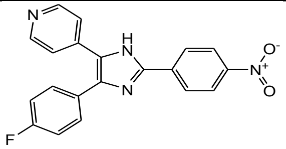 | 4712 |
| 145 | SB220025 | 165806-53-1 | 338.4 | 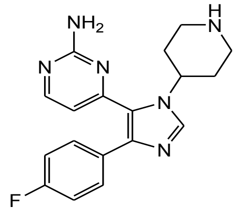 | 5164 |
| 146 | Purvalanol A | 212844-53-6 | 388.9 | 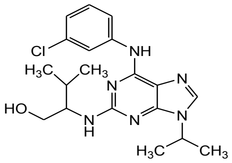 | 4987 |
| 147 | GSK3b Inhibitor XII, TWS119 | | 318.3 | 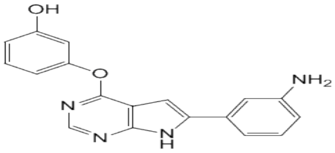 | 9549289 |
| 148 | H-89, Dihydrochloride | 127243-85-0 | 519.3 | 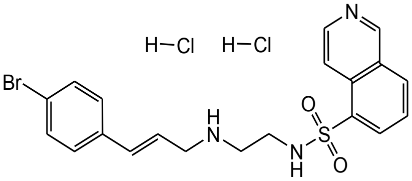 | 5702541 |
| 149 | SB 202474, Negative control for p38 MAPK inhibition studies | | 279.3 | 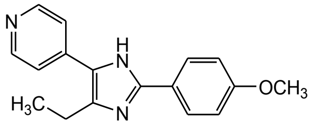 | 5162 |

| | | | | | |
|---|---|---|---|---|---|
| 150 | SB 202190 | 152121-30-7 | 331.3 | 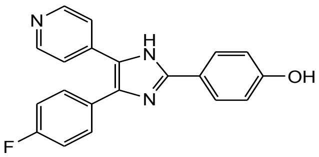 | 5353940 |
| 151 | SB 203580 | 152121-47-6 | 377.4 | 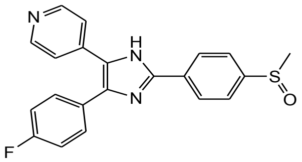 | 176155 |
| 152 | HA 1077, Dihydrochloride<br>Fasudil | 103745-39-7 | 364.3 | 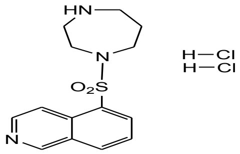 | 16219471 |
| 153 | SB 218078 | 135897-06-2 | 393.4 | 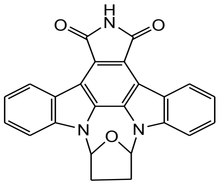 | 3387354 |
| 154 | SC-68376 | 318480-82-9 | 236.3 | 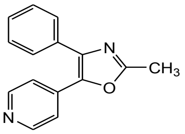 | 5174 |
| 155 | SKF-86002 | 72873-74-6 | 297.4 | 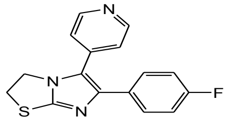 | 5228 |
| 156 | Sphingosine Kinase Inhibitor | | 339.2 | 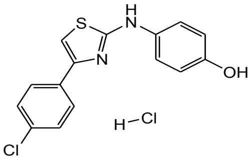 | 16760659 |

| | | | | | |
|---|---|---|---|---|---|
| 157 | Staurosporine, Streptomyces sp. | 62996-74-1 | 466.5 | 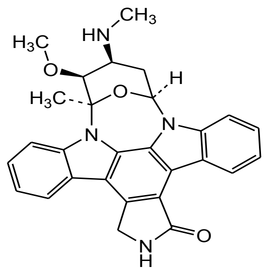 | 451705 |
| 158 | STO-609 | 52029-86-4 | 374.4 | 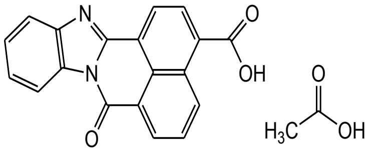 | 16760660 |
| 159 | SU9516 | 666837-93-0 | 241.3 | 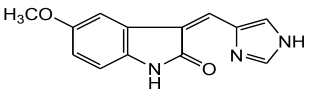 | 5289419 |
| 160 | Tpl2 Kinase Inhibitor | 871307-18-5 | 404.8 | 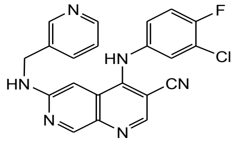 | 9549300 |

| | | | | | |
|---|---|---|---|---|---|
| 161 | Adenosine Kinase Inhibitor | 214697-26-4 | 536.3 | 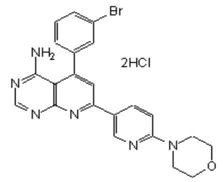 | 26758156 |
| 162 | Akt Inhibitor XII, Isozyme-Selective, Akti-2 | | 747.2 | 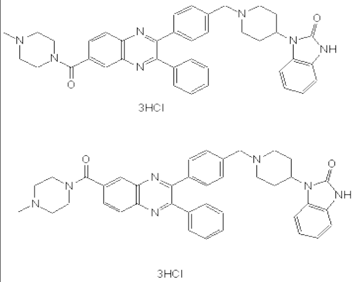 | 56364532 |
| 163 | Arcyriaflavin A, Synthetic | | 325.3 | 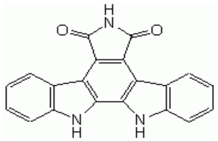 | 26758250 |
| 164 | 1-Azakenpaullone | 676596-65-9 | 328.2 | 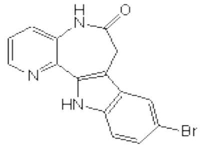 | 26758278 |
| 165 | Bisindolylmaleimide III, Hydrochloride | | 420.9 | 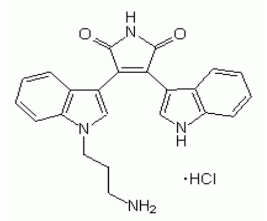 | 26758308 |
| 166 | Bisindolylmaleimide V | 113963-68-1 | 341.3 | 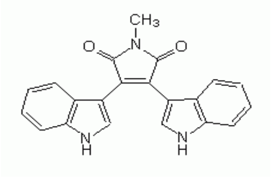 | 26758310 |
| 167 | CR8, (R)-Isomer | | 431.5 | 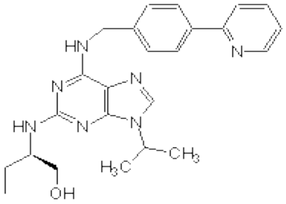 | 58097335 |
| 168 | CR8, (S)-Isomer | | 431.5 | 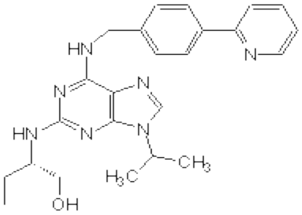 | 81058714 |
| 169 | CaMKII Inhibitor, CK59 | 140651-18-9 | 435.6 | 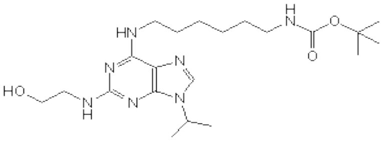 | 56365459 |

| | | | | | |
|---|---|---|---|---|---|
| 170 | Cdk1 Inhibitor IV, RO-3306 | | 351.5 | 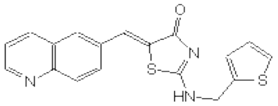 | 26758397 |
| 171 | Cdc7/Cdk9 Inhibitor | | 213.2 | 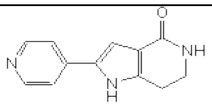 | 81055074 |
| 172 | Casein Kinase II Inhibitor I | 17374-26-4 | 434.7 | 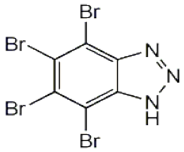 | 26758402 |
| 173 | Casein Kinase II Inhibitor II, DMAT | 749234-11-5 | 476.8 | 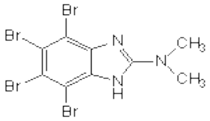 | 26758403 |
| 174 | Keratinocyte Differentiation Inducer | | 429.5 | 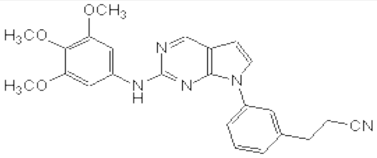 | 56365458 |
| 175 | Cdk2 Inhibitor II | | 395.2 | 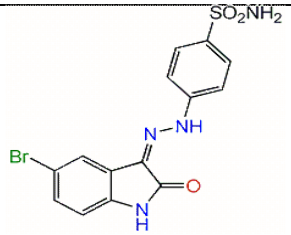 | 26758435 |
| 176 | Cdk2/5 Inhibitor | | 301.8 | 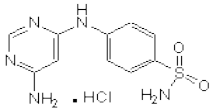 | 26758436 |
| 177 | Cdk Inhibitor, p35 | | 356.4 | 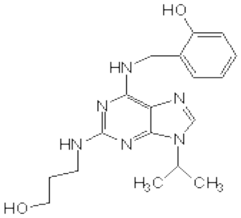 | 26758437 |
| 178 | Chk2 Inhibitor | 724708-21-8 | 295.3 | 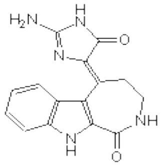 | 26758461 |
| 179 | Compound 401 | 168425-64-7 | 281.3 | 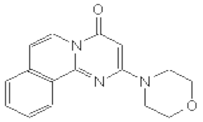 | 56365462 |
| 180 | Cdk2/9 Inhibitor | | 328.4 | 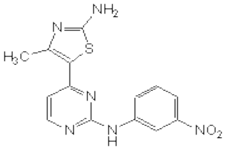 | 26758511 |

| | | | | | |
|---|---|---|---|---|---|
| 181 | Cdk9 Inhibitor II | 140651-18-9 | 218.2 | 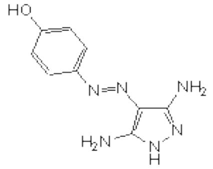 | 26758512 |
| 182 | 4-Cyano-3-methylisoquinoline | | 168.2 | 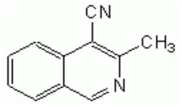 | 26758513 |
| 183 | eEF-2 Kinase Inhibitor, NH125 | 278603-08-0 | 524.6 | 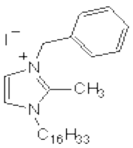 | 81055075 |
| 184 | GSK-3 Inhibitor IX, Control, MeBIO | | 370.2 | 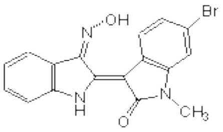 | 26758809 |
| 185 | Gö 7874, Hydrochloride | | 507 | 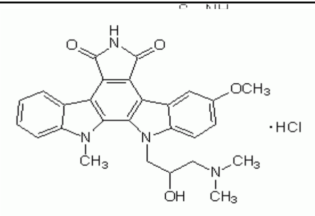 | 26758819 |
| 186 | H-8, Dihydrochloride | 113276-94-1 | 338.3 | 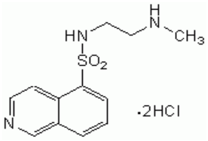 | 26758854 |
| 187 | HA 1004, Dihydrochloride | 92564-34-6 | 366.3 | 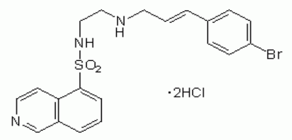 | 26758859 |
| 188 | IKK-2 Inhibitor V | 978-62-1 | 383.7 | 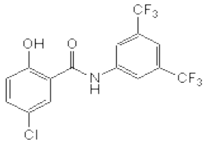 | 26758930 |
| 189 | IKK-2 Inhibitor VI | | 261.3 | 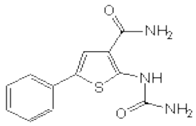 | 26758931 |
| 190 | IKK Inhibitor VII | | 483.6 | 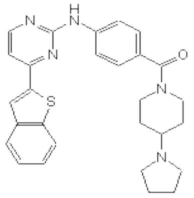 | 26758934 |
| 191 | IKK-2 Inhibitor VIII | | 364.4 | 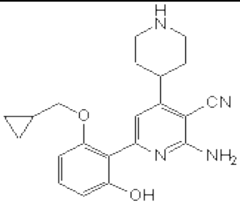 | 26758935 |

| | | | | | |
|---|---|---|---|---|---|
| 192 | IKK-3 Inhibitor IX | | 469.5 | 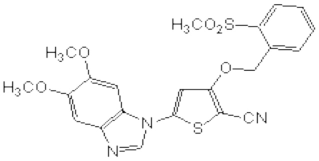 | 81055077 |
| 193 | IKK Inhibitor X | | 322.8 | 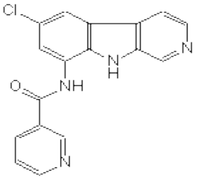 | 81055076 |
| 194 | IKK-2 Inhibitor XI | | 261.3 | 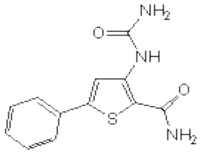 | 58097340 |
| 195 | Indirubin-3′-monoxime, 5-Iodo- | | 403.2 | 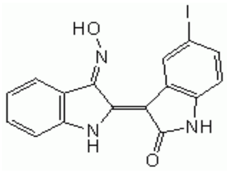 | 26758940 |
| 196 | IP3K Inhibitor | 519178-28-0 | 443.4 | 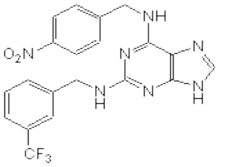 | 26758949 |
| 197 | 5-Iodotubercidin | 24386-93-4 | 392.2 | 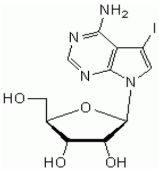 | 26758970 |
| 198 | KT5720 | 108068-98-0 | 537.6 | 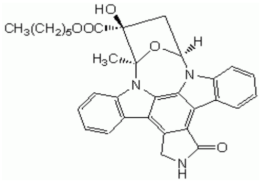 | 26759011 |
| 199 | KN-92 | | 555 | 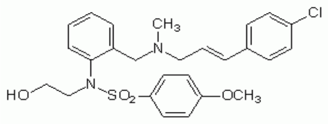 | 26759025 |
| 200 | LY 294002, 4'-$NH_2$ | 942289-87-4 | 322.4 | 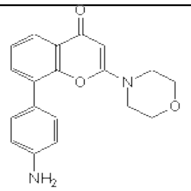 | 57287545 |
| 201 | MEK1/2 Inhibitor II | 212631-61-3 | 476.2 | 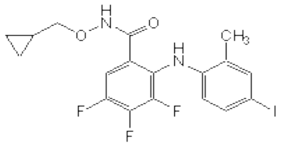 | 56421977 |

| | | | | | |
|---|---|---|---|---|---|
| 202 | MK-2 Inhibitor III | | 358.4 | 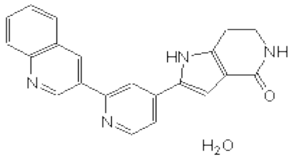 | 57269231 |
| 203 | ML-7, Hydrochloride | 110448-33-4 | 452.7 | 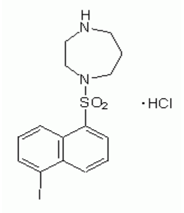 | 26759187 |
| 204 | Necrostatin-1 | 4311-88-0 | 259.3 | 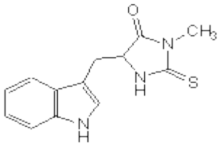 | 26759223 |
| 205 | Olomoucine II | | 370.5 | 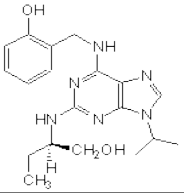 | 26759292 |
| 206 | p21-Activated Kinase Inhibitor III, IPA-3 | | 350.5 | 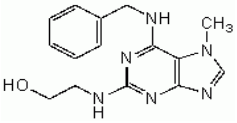 | 57269235 |
| 207 | p38 MAP Kinase Inhibitor IV | 1638-41-1 | 456.9 | 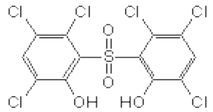 | 58097342 |
| 208 | p38 MAP Kinase Inhibitor VI, JX401 | 349087-34-9 | 355.5 | 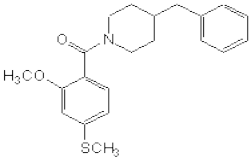 | 81055078 |
| 209 | p38 MAP Kinase Inhibitor VII, SD-169 | 1670-87-7 | 160.2 | 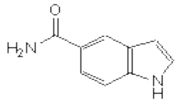 | 57269236 |
| 210 | p38 MAP Kinase Inhibitor VIII | 321351-00-2 | 415.7 | 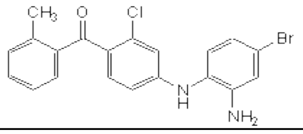 | 57287541 |
| 211 | PIKfyve Inhibitor | 371942-69-7 | 467.5 | 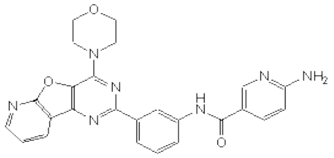 | 81055079 |
| 212 | PIM1 Kinase Inhibitor II | | 367.2 | 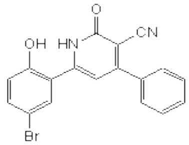 | 81055080 |

| | | | | | |
|---|---|---|---|---|---|
| 213 | PIM1 Kinase Inhibitor IV | | 322.1 | 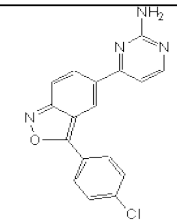 | 56459315 |
| 214 | PIM1/2 Kinase Inhibitor V | | 273.2 | 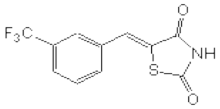 | 81055081 |
| 215 | PIM1/2 Kinase Inhibitor VI | | 263.3 | 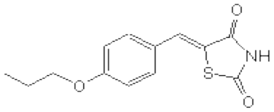 | 81055082 |
| 216 | PI 3-Kα Inhibitor IV | | 386.3 | 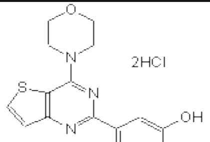 | 26759375 |
| 217 | PI 3-Kγ/CKII Inhibitor | | 305.3 | 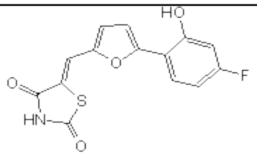 | 26759376 |
| 218 | PI 3-Kβ Inhibitor VI, TGX-221 | 663619-89-4 | 364.4 | 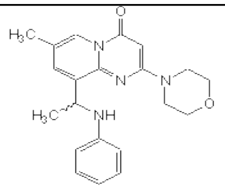 | 81055083 |
| 219 | PI 3-Kγ Inhibitor VII | | 249.2 | 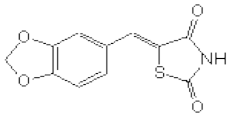 | 56365803 |
| 220 | PI 3-Kα Inhibitor VIII | | 524.8 | 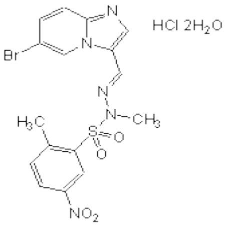 | 81055084 |
| 221 | Polo-like Kinase Inhibitor I | | 487.6 | 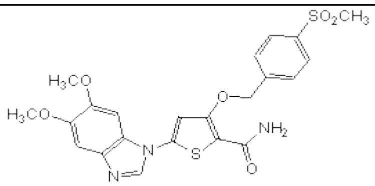 | 81055085 |
| 222 | Polo-like Kinase Inhibitor II, BTO-1 | 40647-02-7 | 264.2 | 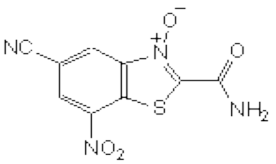 | 58097343 |

| | | | | | |
|---|---|---|---|---|---|
| 223 | UCN-01 | 112953-11-4 | 482.5 | 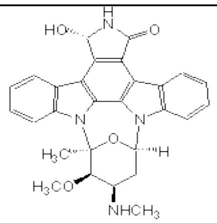 | 81055086 |
| 224 | Quercetagetin | 90-18-6 | 318.3 | 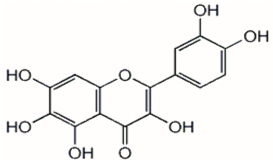 | 26759427 |
| 225 | Ras/Rac Transformation Blocker, SCH 51344 | 171927-40-5 | 316.4 | 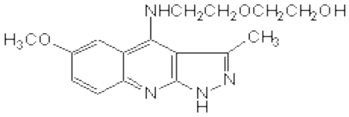 | 26759438 |
| 226 | Reversine | 656820-32-5 | 393.5 | 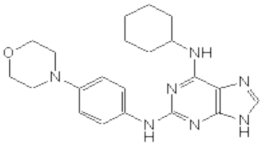 | 26759441 |
| 227 | Rho Kinase Inhibitor | | 392.3 | 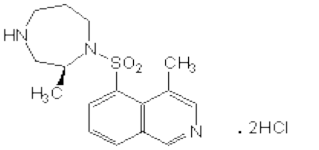 | 26759444 |
| 228 | Rho Kinase Inhibitor II | | 316.6 | 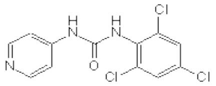 | 26759445 |
| 229 | Rho Kinase Inhibitor V | | 321.3 | 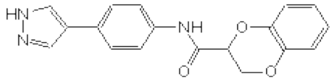 | 81055087 |
| 230 | Roscovitine | 186692-46-6 | 354.5 | 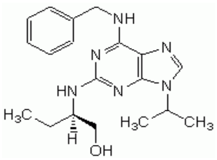 | 26759456 |
| 231 | Roscovitine, (S)-Isomer | | 354.5 | 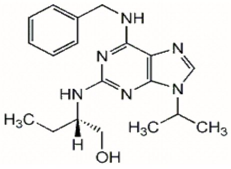 | 26759457 |
| 232 | Ro-31-8220 | 138489-18-6 | 553.7 | 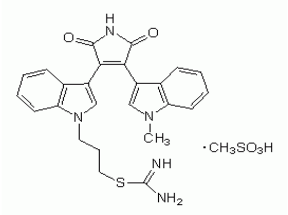 | 26759466 |
| 233 | RSK Inhibitor, SL0101 | | 516.5 | 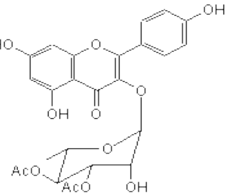 | 81058712 |

| | | | | | |
|---|---|---|---|---|---|
| 234 | SB 203580, Sulfone | 152121-46-5 | 393.4 | 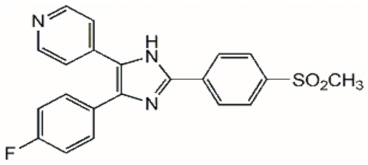 | 26759484 |
| 235 | SB 239063 | | 368.4 | 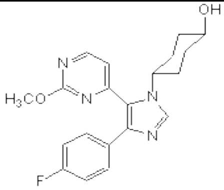 | 26759488 |
| 236 | Scytonemin, *Lyngbya* sp. | | 544.6 | 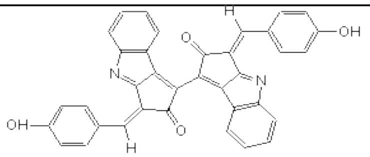 | 26759499 |
| 237 | Stem-Cell Factor/c-Kit Inhibitor, ISCK03 | | 355.5 | 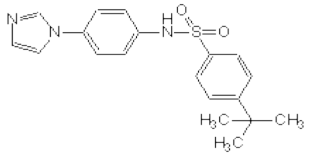 | 81058713 |
| 238 | Ste11 MAPKKK Activation Inhibitor | | 350.2 | 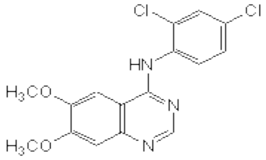 | 81058715 |
| 239 | Tpl2 Kinase Inhibitor II | | 390.5 | 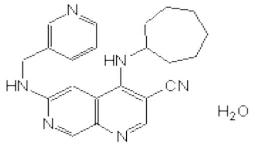 | 57287543 |
| 240 | TX-1918 | | 228.2 | 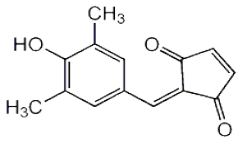 | 26759674 |
| 241 | WHI-P180, Hydrochloride | 211555-08-7 | 297.3 | 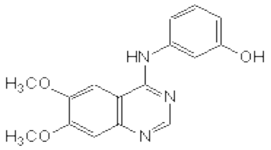 | 57269238 |
| 242 | Wee1/Chk1 Inhibitor | | 346.3 | 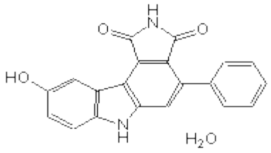 | 26759753 |
| 243 | Wee1 Inhibitor | | 362.8 | 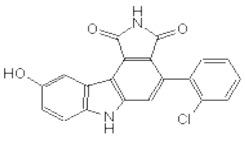 | 26759754 |
| 244 | Wee1 Inhibitor II | | 418.9 | 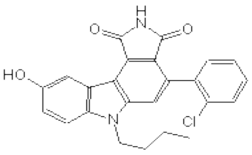 | 26759755 |
