# Supplementary material for: A Comparison of Protein Kinases Inhibitor Screening Methods Using Both Enzymatic Activity and Binding Affinity Determination
Source: PLoS One. 2014 Jun 10;9(6):e98800. doi: 10.1371/journal.pone.0098800 (PMC4051630; doi:10.1371/journal.pone.0098800)
Supplement: Table S2 — Melting temperature (Tm) shift data, expressed in degrees Celcius, for each compound versus kinase. Average of 3 experiments, each in duplicate. (PDF) [file pone.0098800.s002.pdf]

Table S2

Compound Name	Pubchem Compound ID	AKT3 Catalytic Domain	CDK2 + Cyclin A	CDK2 + Cyclin E	CHK2 Catalytic Domain	DMPK Catalytic Domain	FAK2 Catalytic Domain	FES Catalytic Domain+ SH2	FES Catalytic Domain	PAK4 Catalytic Domain+ PBD	PAK4 Catalytic Domain	PAK7 Catalytic Domain	SRC Catalytic Domain	SRC Catalytic Domain+ SH2+SH3	STK3 Catalytic domain+S ARAH	STK3 Catalytic Domain
5-Iodotubercidin	97297	0.0	2.5	1.3	0.6	0.2	0.4	0.0	1.0	0.7	0.7	0.4	-0.3	0.1	1.5	3.0
Akt Inhibitor IV	5719375	-1.2	-2.0	-1.9	-1.1	-1.1	-1.2	-0.8	-0.6	-1.0	-2.2	-0.6	-0.5	-0.7	-0.4	-0.6
Akt Inhibitor VIII, Isozyme-Selective, Akti-1/2	10196499	-0.2	0.2	-0.1	0.4	-0.4	0.1	-0.1	-0.2	-0.3	-0.1	0.0	-0.2	-0.3	-0.1	-0.1
Akt Inhibitor XII, Isozyme-Selective, Akti-2	10196499	-0.3	-0.2	-0.4	-0.1	-0.6	-0.2	-0.4	-0.1	0.0	-0.3	0.0	0.0	-0.1	0.3	-0.1
Aloisine A, RP107	5326843	0.1	2.5	1.4	2.7	1.4	1.2	0.5	0.9	1.3	1.3	1.2	1.5	1.1	1.4	1.6
Alsterpaullone	5005498	0.1	0.4	0.2	0.9	1.8	0.4	0.1	0.3	0.3	0.2	0.3	0.0	0.2	0.7	0.3
Alsterpaullone, 2-Cyanoethyl	16760286	0.1	0.5	0.5	1.9	3.4	0.6	0.3	1.3	0.3	0.9	1.3	0.2	0.3	1.5	1.7
Aminopurvalanol A	6604931	0.1	4.7	3.4	1.1	1.7	2.5	1.0	1.8	9.4	5.3	4.4	3.1	2.6	1.6	2.4
AMPK Inhibitor, Compound C	11524144	0.3	1.0	0.5	1.1	1.5	0.1	0.4	1.2	0.8	0.7	0.5	0.5	0.6	0.4	0.3
Arcyriaflavin A, Synthetic	5327723	0.0	-0.2	-0.4	-1.7	-1.1	-0.5	-0.3	-0.1	0.5	0.2	-0.1	-0.3	-0.2	-2.3	-0.6
Aurora Kinase Inhibitor II	6610278	0.1	0.1	-0.1	7.0	0.1	0.3	0.2	0.1	0.1	-0.2	-0.1	0.7	0.4	0.4	0.9
Aurora Kinase Inhibitor III	9549303	0.1	0.2	-0.1	0.4	0.9	0.2	0.3	0.2	1.1	0.8	0.0	0.2	0.0	0.4	0.1
Aurora Kinase/Cdk Inhibitor	16760303	0.0	6.4	4.3	6.5	4.0	1.7	0.8	1.9	2.7	2.5	2.2	0.4	0.3	4.9	5.7
Bisindolylmaleimide I	2396	0.4	1.8	0.9	4.6	3.9	0.5	0.6	0.6	0.6	1.0	0.6	0.4	0.0	2.9	1.0
Bisindolylmaleimide III, Hydrochloride	16760314	0.6	2.1	0.7	5.6	4.2	0.7	0.5	0.8	1.6	1.4	1.1	-0.2	0.0	4.6	1.8
Bisindolylmaleimide IV	2399	0.0	0.9	0.7	4.1	2.7	0.1	0.9	0.5	0.0	0.4	0.1	0.3	0.0	2.0	0.9
Casein Kinase II Inhibitor I	1694	0.1	0.8	0.8	2.2	0.6	0.1	0.8	0.0	0.9	0.2	-0.1	-0.1	0.2	0.4	1.1
Casein Kinase II Inhibitor II, DMAT	5326976	-0.2	0.5	-0.2	0.3	0.4	3.2	-0.1	-0.1	0.3	0.2	4.1	-0.2	0.9	0.4	0.3
Cdc2-Like Kinase Inhibitor, TG003	1893668	-0.1	0.0	0.0	0.0	0.2	0.1	-0.3	0.0	-0.3	-0.1	0.0	-0.1	-0.1	-0.2	0.0
Cdk Inhibitor, p35	4155347	-0.1	3.6	2.0	0.8	0.3	0.0	0.2	1.1	3.9	1.4	1.1	-0.3	-0.1	0.4	0.3
Cdk/Crk Inhibitor	9549301	0.0	7.5	5.5	0.6	1.3	0.5	0.1	0.6	1.1	1.9	2.0	-0.2	-0.2	0.2	0.8
Cdk1 Inhibitor IV, RO-3306	11631681	-0.1	0.9	0.0	1.7	2.8	0.3	0.0	0.1	0.9	0.4	0.1	-0.1	0.0	0.6	0.2
Cdk1 Inhibitor, CGP74514A	2794188	0.0	4.4	3.7	1.8	1.4	1.9	0.7	1.6	8.1	5.6	5.4	1.2	1.3	1.2	1.3
Cdk1/2 Inhibitor III	5330812	0.3	2.9	4.4	7.3	4.5	2.4	2.9	6.8	4.9	5.9	5.0	1.0	1.3	10.5	9.5
Cdk2 Inhibitor II	5858639	-0.1	0.4	1.7	0.1	0.3	0.0	-0.2	-0.2	0.7	0.2	0.1	-0.2	0.1	2.8	1.0
Cdk2 Inhibitor III	6918386	-0.1	2.9	1.5	1.9	-0.3	-0.5	0.9	2.4	1.2	1.4	1.2	-0.1	-0.2	-0.1	0.1

Cdk2 Inhibitor IV, NU6140	10202471	-0.1	1.5	0.2	4.0	2.0	2.4	0.6	2.8	2.9	5.1	4.7	1.8	1.3	2.5	1.9
Cdk2/9 Inhibitor	447961	-0.1	1.3	1.4	1.8	0.4	0.5	0.1	0.3	2.0	0.7	0.3	0.5	0.6	0.4	0.7
Cdk4 Inhibitor	5330797	-0.3	-0.2	0.7	N.A	0.8	-0.1	-0.5	-0.3	0.4	0.7	-0.3	-1.1	-0.6	-1.6	-0.9
Cdk4 Inhibitor III	481747	-0.2	0.0	0.5	-0.6	-0.3	-0.1	-0.2	-1.0	-0.1	0.1	-0.1	-0.2	-0.2	-0.1	-0.1
Chk2 Inhibitor	16760370	0.0	2.9	1.9	8.7	1.4	2.6	0.6	3.0	0.6	0.7	0.3	0.2	0.3	3.4	2.3
Chk2 Inhibitor II	9969021	0.1	0.1	-0.1	0.6	-0.3	-0.1	0.0	0.2	0.0	0.1	0.0	-0.3	-0.1	-0.1	0.1
Compound 52	2856	0.0	2.3	1.7	1.1	0.0	0.9	0.6	2.3	5.0	2.0	1.8	0.9	0.8	0.7	1.2
CR8, (R)-Isomer	58097335	-0.1	2.8	0.9	0.8	-0.2	0.2	0.2	1.6	2.8	2.5	2.4	0.0	-0.2	0.4	0.2
CR8, (S)-Isomer	81058714	-0.1	2.4	1.3	0.8	-0.4	0.2	0.5	1.7	1.2	2.0	2.0	-0.1	-0.2	0.4	0.2
Diacylglycerol Kinase Inhibitor II	657356	-0.2	-1.4	-0.9	-1.9	-5.2	-0.1	-0.1	0.7	0.2	-0.4	-0.4	-0.8	-0.5	-0.8	-0.9
DNA-PK Inhibitor V	16760391	0.1	0.0	0.2	0.2	0.6	0.3	0.3	0.1	0.5	0.4	0.0	0.2	0.0	0.5	0.0
eEF-2 Kinase Inhibitor, NH125	10436839	-1.1	-3.4	-3.6	N.A.	N.A.	-0.6	-2.5	-2.1	-4.6	-3.1	-1.6	-1.2	-1.4	-0.8	-0.2
EGFR/ErbB-2 Inhibitor	9843206	0.1	0.0	0.1	11.2	0.3	-0.1	0.1	0.0	0.0	0.3	0.0	1.4	0.8	0.2	0.2
EGFR/ErbB-2/ErbB-4 Inhibitor	11566580	0.0	-0.3	-0.2	0.3	-0.7	-0.2	-0.1	0.0	0.1	-0.5	0.0	-0.2	0.0	-0.1	0.2
Fascaplysin, Synthetic	73292	0.1	-0.2	-0.2	-0.1	0.4	-0.2	0.0	0.2	-0.4	-0.2	-0.1	-0.3	-0.2	-0.5	-0.2
Flt-3 Inhibitor II	11601743	0.1	0.9	0.2	2.2	-0.2	0.4	0.0	1.0	0.9	0.9	0.6	1.3	1.3	2.3	2.3
Gö 6976	3501	0.0	0.4	-0.2	0.9	0.0	2.5	-0.1	0.4	4.6	1.9	1.0	0.1	-0.4	6.1	5.9
Gö 6983	3499	0.0	0.4	0.1	5.6	1.9	-0.2	0.7	0.9	-0.1	0.0	0.1	0.2	-0.1	0.7	0.2
Gö 7874, Hydrochloride	26758819	0.2	0.2	-0.5	1.1	0.2	0.7	0.0	0.3	0.1	-0.2	0.1	-0.3	-0.2	2.2	1.4
GSK-3 Inhibitor IX	5287844	0.3	-0.3	0.6	1.2	0.5	0.5	-0.4	0.1	2.4	1.4	0.1	1.0	1.3	3.6	2.7
GSK-3 Inhibitor IX, Control, MeBIO	6538821	-0.1	-0.3	-0.3	-0.3	-0.1	0.1	-0.1	-0.1	0.3	0.0	0.0	0.0	0.0	0.4	0.1
GSK-3 Inhibitor X	6538818	0.1	-0.5	-0.2	-0.1	0.5	-0.5	0.0	0.4	1.0	0.6	0.1	-0.1	-0.5	0.5	-0.1
GSK-3 Inhibitor XIII	6419766	0.2	0.3	-0.1	1.9	0.5	0.8	0.2	1.0	0.8	3.0	2.0	1.3	1.6	0.9	3.7
GSK3b Inhibitor XII, TWS119	9549289	0.1	0.7	0.2	2.1	1.2	1.5	2.0	4.1	1.5	1.3	1.1	2.6	3.1	0.5	0.3
H-89, Dihydrochloride	5702541	0.3	0.5	0.3	0.5	0.0	0.8	0.2	0.4	0.0	0.4	0.3	-0.1	0.0	0.4	0.0
IKK Inhibitor VII	9549298	0.2	0.7	0.5	1.9	1.2	1.8	1.4	3.3	0.5	1.7	1.8	0.6	0.5	1.2	0.7
IKK-2 Inhibitor IV	9903786	0.1	0.7	0.4	3.6	1.8	1.2	0.5	1.5	2.4	1.5	1.3	1.2	1.0	1.6	2.8
IKK-2 Inhibitor V	5081913	-0.3	-0.7	0.5	-1.0	-0.1	0.1	0.2	-0.6	0.7	0.5	0.0	0.0	0.4	-0.1	0.2
IKK-2 Inhibitor VI	6419765	0.0	0.8	0.2	3.6	1.4	1.4	0.7	1.8	2.5	1.3	1.3	1.3	1.1	2.0	2.4
IKK-2 Inhibitor XI	10220851	0.0	0.9	0.7	5.8	1.6	1.5	1.1	2.1	2.9	1.7	1.3	1.5	1.0	1.4	2.4
Indirubin Derivative E804	6419764	0.7	-0.2	1.1	2.2	0.6	2.8	1.0	1.1	2.1	1.9	1.7	3.1	2.8	3.5	5.2

Indirubin-3′-monoxime	5326739	0.2	-0.1	0.1	1.1	0.6	-0.1	0.1	0.2	1.2	0.8	0.2	0.2	0.4	1.0	2.4
Indirubin-3′-monoxime, 5-Iodo-	5472313	-0.1	-0.8	-0.2	-0.1	-0.2	-0.3	-0.1	-0.1	2.3	0.3	-0.1	-0.3	-0.6	5.6	1.2
Isogranulatimide	6419741	2.9	0.1	0.2	4.5	0.5	0.8	0.5	1.6	-0.2	0.4	0.2	-0.1	-0.2	1.3	2.6
JAK Inhibitor I	5494425	-0.1	0.0	0.0	8.8	-0.5	3.0	0.1	0.1	0.2	0.2	0.2	-0.1	-0.1	3.2	2.1
JAK3 Inhibitor VI	16760524	0.0	2.3	0.9	3.0	0.8	0.2	0.1	1.1	1.1	0.6	0.7	-0.4	-0.1	5.7	2.7
JNK Inhibitor II	8515	0.1	1.2	0.3	5.2	1.4	1.0	0.9	2.6	1.0	1.1	0.7	-0.3	-0.1	1.3	2.0
JNK Inhibitor V	11422035	0.0	0.2	0.3	0.3	0.8	0.4	0.0	-0.1	0.1	0.1	0.0	-0.2	0.1	0.3	0.2
K-252a, Nocardiopsis sp.	490561	3.2	-1.2	-0.1	9.6	3.4	3.0	1.9	3.8	2.5	7.3	5.9	0.7	1.0	5.4	4.8
Kenpaullone	3820	0.0	0.4	0.3	3.4	1.9	0.3	0.0	0.4	3.6	1.4	0.7	0.4	0.2	1.5	0.7
Keratinocyte Differentiation Inducer	11453158	0.0	4.3	2.3	8.3	3.2	-0.1	3.4	5.5	7.3	5.5	0.0	3.0	1.8	4.3	3.6
Lck Inhibitor	6603792	-0.6	-0.7	-1.0	2.2	-1.5	-0.4	0.3	0.7	-0.6	-0.7	0.0	9.4	6.4	0.2	1.3
MEK Inhibitor II	389898	0.3	0.1	-0.3	0.0	-0.2	-0.1	0.0	0.0	-0.3	0.0	0.0	-0.2	0.0	0.1	-0.1
Met Kinase Inhibitor	9549297	0.0	0.4	0.1	1.2	1.2	0.1	0.1	0.6	0.3	0.9	0.2	0.3	0.2	0.9	0.7
MK-2 Inhibitor III	57269231	0.0	0.1	-0.6	1.2	0.3	0.1	-0.2	-0.1	0.1	0.0	0.0	-0.1	0.1	0.4	0.0
Olomoucine II	5494414	-0.1	3.3	2.2	0.2	-0.5	0.2	0.1	0.7	3.5	1.3	1.1	-0.1	-0.2	-0.1	0.3
PDGF Receptor Tyrosine Kinase Inhibitor II	5330548	-0.1	-0.4	-1.0	1.4	-0.6	0.3	0.1	0.3	-0.3	0.0	0.1	1.1	0.6	1.2	0.5
PDGF RTK Inhibitor	16760609	-0.3	-0.9	-1.1	7.6	-1.7	1.0	1.5	1.6	-0.9	-0.8	-0.1	5.9	3.9	0.0	1.7
PDK1/Akt/Flt Dual Pathway Inhibitor	5113385	3.1	0.1	0.4	0.6	-0.1	-0.8	-0.1	0.0	-0.2	-0.5	-0.2	-0.2	-0.7	0.0	-0.2
PI 3-Kg Inhibitor	5289247	0.0	0.2	-0.1	0.4	0.8	-0.5	0.1	0.1	-0.2	0.1	0.0	-0.2	0.0	0.1	0.1
PI 3-Kα Inhibitor VIII	81055084	1.3	4.2	0.7	2.5	3.1	1.6	1.4	3.1	2.1	3.0	1.8	0.7	0.9	4.2	2.7
PI 3-Kγ Inhibitor VII	233033	0.0	-0.2	0.1	0.1	0.3	0.0	-0.5	0.0	-0.1	-0.1	-0.1	-0.5	-0.2	-0.2	0.2
PI 3-Kγ/CKII Inhibitor	26759376	-0.3	0.2	0.2	2.5	0.3	0.1	0.5	-0.6	1.3	0.9	-0.1	0.0	0.1	0.5	0.3
PIM1 Kinase Inhibitor II	1235170	-0.1	0.4	0.3	1.9	0.1	0.2	0.4	-0.1	0.5	0.1	0.0	0.0	0.2	0.2	0.5
PIM1/2 Kinase Inhibitor V	2864586	0.1	0.3	0.2	0.5	0.1	0.2	0.4	0.5	0.4	0.2	0.1	0.1	0.2	0.4	0.2
PKCb Inhibitor	6419755	-0.1	0.5	0.0	2.0	1.6	-0.5	-0.1	0.4	-0.1	-0.2	0.0	0.1	-0.3	1.7	0.3
PKR Inhibitor	6490494	0.3	4.4	4.3	4.0	0.8	1.1	0.7	3.2	1.8	1.9	1.9	0.7	0.3	7.0	4.9
PKR Inhibitor, Negative Control	16760619	-0.3	-0.5	0.6	3.9	3.2	-0.2	0.5	0.4	1.5	0.9	0.3	-0.4	-0.4	2.1	1.2
Purvalanol A	4987	-0.1	1.7	3.2	0.0	0.4	1.7	0.2	0.5	7.4	3.2	1.6	1.1	1.0	1.0	1.1
Quercetagetin	5281680	2.5	0.2	0.7	1.1	-1.5	0.2	0.1	0.1	0.3	0.0	0.1	-0.1	0.0	0.5	0.2
Reversine	210332	-0.2	0.7	0.8	4.7	3.4	3.6	1.5	4.5	4.2	4.3	3.3	3.1	2.6	3.4	3.5
Rho Kinase Inhibitor V	25093233	0.1	0.1	0.9	0.7	0.2	-0.1	-0.2	-0.1	-0.2	0.3	0.0	-0.1	-0.1	0.3	-0.2

Ro-31-8220	5083	1.6	-2.2	N.A	6.6	4.8	0.5	0.8	1.0	1.6	1.5	0.9	0.2	0.0	2.9	1.8
Roscovitine, (S)-Isomer	6603989	0.0	2.6	2.0	0.6	0.3	0.2	0.9	1.2	2.6	1.4	0.7	-0.4	-0.2	0.0	0.0
SB 218078	3387354	16.8	-1.6	0.0	24.9	15.4	19.6	-1.3	16.0	-0.6	2.5	0.5	1.9	10.3	0.6	14.2
Scytonemin, *Lyngbya* sp.	5486761	0.0	0.3	0.8	-0.1	0.3	0.2	0.2	-0.1	0.7	0.6	0.0	0.0	0.3	0.4	0.5
Src Kinase Inhibitor I	1474853	-0.1	0.0	0.2	3.0	0.7	0.6	0.1	0.1	-0.1	-0.2	0.0	2.1	1.6	0.1	0.3
Staurosporine, N-benzoyl-	16760627	0.9	0.4	-0.3	0.5	1.7	3.4	0.7	1.9	1.2	0.9	0.5	1.2	1.1	6.9	4.1
Staurosporine, Streptomyces sp.	451705	5.7	2.5	1.9	11.3	8.4	9.2	6.3	8.2	10.9	10.5	9.4	3.5	3.6	15.1	10.7
Ste11 MAPKKK Activation Inhibitor	1474860	-0.2	0.1	-0.1	10.6	0.4	0.4	0.1	0.1	0.1	0.0	0.0	0.5	0.7	0.6	0.3
SU11652	5329103	N.A.	0.6	0.5	4.8	1.9	1.3	0.9	2.8	2.0	2.8	-6.7	1.2	0.5	3.8	-1.0
SU9516	5289419	0.2	3.7	3.3	2.7	1.4	1.0	0.5	1.1	2.0	1.7	1.2	0.3	0.3	6.2	4.5
Syk Inhibitor	6419747	0.0	0.7	0.4	5.2	0.9	0.4	0.4	2.0	2.2	1.7	0.9	0.1	0.2	2.0	2.0
Syk Inhibitor II	16760670	0.0	0.5	0.6	4.7	0.7	1.9	1.3	3.1	4.8	4.7	5.1	0.1	0.4	1.3	1.6
Tpl2 Kinase Inhibitor	9549300	0.1	0.2	0.5	0.3	1.4	0.5	0.2	0.0	0.6	0.5	0.1	0.2	0.1	0.4	0.2
TX-1918	6419746	4.5	0.4	0.4	2.6	-2.8	0.5	-0.1	-0.1	1.1	-1.0	-6.6	0.0	-1.2	-0.1	0.1
UCN-01	72271	3.2	2.7	0.9	1.7	5.9	5.9	1.3	5.2	6.8	6.2	7.4	0.4	1.2	12.7	7.6
VEGF Receptor 2 Kinase Inhibitor I	6419834	0.3	-0.1	0.2	1.1	0.7	0.5	0.1	0.3	0.4	0.6	-0.1	0.0	0.0	0.9	-0.4
VEGF Receptor 2 Kinase Inhibitor III	5329098	0.2	0.1	0.5	5.7	1.1	2.8	0.4	1.0	1.3	0.9	0.1	0.4	0.1	1.5	N.A
Wee1 Inhibitor	10384072	0.2	0.5	1.1	4.6	3.1	2.5	1.9	3.1	2.5	2.3	2.1	0.4	0.5	4.2	4.1
Wee1 Inhibitor II	10319891	0.1	0.1	0.8	0.1	0.6	0.9	0.2	0.0	1.0	0.5	0.3	0.3	0.2	1.2	-0.1
Wee1/Chk1 Inhibitor	16760707	0.1	0.2	0.8	5.9	2.2	0.3	0.2	0.2	1.5	0.7	1.0	0.1	0.1	3.7	1.1
WHI-P180, Hydrochloride	5687	0.0	0.4	-0.1	3.0	0.9	0.5	0.7	0.6	0.4	0.5	0.2	0.7	1.2	1.3	0.3
