# Supplementary material for: A Comparison of Protein Kinases Inhibitor Screening Methods Using Both Enzymatic Activity and Binding Affinity Determination
Source: PLoS One. 2014 Jun 10;9(6):e98800. doi: 10.1371/journal.pone.0098800 (PMC4051630; doi:10.1371/journal.pone.0098800)
Supplement: Table S3 — IC50 values, expressed in µM, measured for the 32 most potent compounds for each kinase. Average of three experiments. (PDF) [file pone.0098800.s003.pdf]

Table S3

Compound Name	Pubchem Compound ID	AKT3 Catalytic Domain	CDK2 + Cyclin A	CDK2 + Cyclin E	CHK2 Catalytic Domain	DMPK Catalytic Domain	FAK2 Catalytic Domain	FES Catalytic Domain	FES Catalytic Domain+ SH2	PAK4 Catalytic Domain	PAK4 Catalytic Domain+ PBD	PAK7 Catalytic Domain	SRC Catalytic Domain	SRC Catalytic Domain+ SH2+SH3	STK3 Catalytic Domain	STK3 Catalytic domain+SARAH
5-Iodotubercidin	97297														21.197	18.297
Akt Inhibitor IV	5719375	7.365								>25	>25					
Akt Inhibitor VIII, Isozyme-Selective, Akti-1/2	10196499	>25								>25	>25					
Akt Inhibitor XII, Isozyme-Selective, Akti-2	10196499	>25														
Aloisine A, RP107	5326843		1.722	1.046									1.677	2.080		
Alsterpaullone	5005498				0.140		2.040	0.648	5.832			0.926				
Alsterpaullone, 2-Cyanoethyl	16760286		0.216	0.333	0.133		0.447	0.200	1.544	0.130	0.158	0.291				
Aminopurvalanol A	6604931		0.359	0.137	4.983		0.809	1.986	10.277	0.069	0.139	0.351	0.073	0.077	4.937	4.272
AMPK Inhibitor, Compound C	11524144												1.468	1.931		
Arcyriaflavin A, Synthetic	5327723				0.110											
Aurora Kinase Inhibitor II	6610278				1.392											
Aurora Kinase Inhibitor III	9549303							4.364	>25							
Aurora Kinase/Cdk Inhibitor	16760303		0.119	0.132								2.218				
Bisindolylmaleimide I	2396	2.568			3.728	0.125				>25	19.700				1.503	1.393
Bisindolylmaleimide III, Hydrochloride	16760314	1.204			1.683	0.025	3.969			3.398	2.833				0.408	0.255
Bisindolylmaleimide IV	2399					0.812									2.877	1.439
Casein Kinase II Inhibitor I	1694					5.415										
Casein Kinase II Inhibitor II, DMAT	5326976						0.042	0.094	0.434			0.136				
Cdc2-Like Kinase Inhibitor, TG003	1893668					2.232										
Cdk Inhibitor, p35	4155347		1.211	0.506												
Cdk/Crk Inhibitor	9549301		0.067	0.141								2.690				
Cdk1 Inhibitor IV, RO-3306	11631681		9.735	3.309		2.597										

Cdk1 Inhibitor, CGP74514A	2794188					6.478				0.245	0.366	0.330				
Cdk1/2 Inhibitor III	5330812		0.056	0.164	0.297	>25	0.350	0.095	0.715	0.072	0.087	0.222	0.358	0.635	0.020	0.037
Cdk2 Inhibitor II	5858639		0.448	0.263												
Cdk2 Inhibitor III	6918386		2.544	1.145				5.583	24.535							
Cdk2 Inhibitor IV, NU6140	10202471		6.471	4.493			0.565	1.434	8.081	0.154	0.304	0.539				
Cdk2/9 Inhibitor	447961												0.604	0.819		
Cdk4 Inhibitor	5330797	4.768				0.100							13.621	>25	2.939	1.653
Cdk4 Inhibitor III	481747	0.297														
Chk2 Inhibitor	16760370		3.592	1.275	0.109		0.644	2.070	11.024							
Chk2 Inhibitor II	9969021				0.029											
Compound 52	2856							2.857	15.349			2.553				
CR8, (R)-Isomer	58097335		1.869	0.529								4.293				
CR8, (S)-Isomer	81058714							4.949	24.565			6.735				
Diacylglycerol Kinase Inhibitor II	657356									>25	>25					
DNA-PK Inhibitor V	16760391									>25	>25					
eEF-2 Kinase Inhibitor, NH125	10436839	2.207	3.910	24.484	3.436	3.871	2.786	3.528	>25	5.613	6.466				>25	7.000
EGFR/ErbB-2 Inhibitor	9843206				0.438											
EGFR/ErbB-2/ErbB-4 Inhibitor	11566580									>25	>25					
Fascaplysin, Synthetic	73292	0.460														
Flt-3 Inhibitor II	11601743				11.019											
Gö 6976	3501						0.046					0.097			0.045	0.052
Gö 6983	3499				3.965	0.587		4.661	>25						6.395	4.341
Gö 7874, Hydrochloride	26758819	0.848				0.141									0.290	0.194
GSK-3 Inhibitor IX	5287844		1.105	0.152			0.393			0.223	0.328	0.644	0.049	0.072		
GSK-3 Inhibitor IX, Control, MeBIO	6538821									>25	>25					

GSK-3 Inhibitor X	6538818	4.867	78.280	0.787									0.048	0.102		
GSK-3 Inhibitor XIII	6419766				1.930		0.672			0.082	0.096	0.305	0.144	0.146		
GSK3b Inhibitor XII, TWS119	9549289							0.339	2.190				0.038	0.043		
H-89, Dihydrochloride	5702541	4.590														
IKK Inhibitor VII	9549298						1.599	0.487	4.366	1.800	1.200	2.182	1.261	1.367		
IKK-2 Inhibitor IV	9903786											4.674				
IKK-2 Inhibitor V	5081913									>25	>25					
IKK-2 Inhibitor VI	6419765												1.183	1.468	6.368	4.289
IKK-2 Inhibitor XI	10220851				0.747			2.530	12.378			2.918	1.526	1.987	7.751	4.884
Indirubin Derivative E804	6419764		2.115	0.930			0.316					1.331	0.231	0.272	0.198	0.181
Indirubin-3’-monoxime	5326739				0.883		1.989						1.044	1.108	1.777	0.556
Indirubin-3’-monoxime, 5-Iodo-	5472313	9.361	1.520	0.379								0.078	0.645	1.306	0.073	0.110
Isogranulatimide	6419741	3.595				0.342	1.744	13.173	>25						5.227	3.831
JAK Inhibitor I	5494425				0.098		0.222									
JAK3 Inhibitor VI	16760524		0.123	0.296	0.843	>25									0.063	0.033
JNK Inhibitor II	8515				1.366			1.798	8.293							
JNK Inhibitor V	11422035	4.846				1.216										
K-252a, Nocardiopsis sp.	490561	0.378	0.332	0.517		0.819	0.378	0.285	1.411	0.019	0.025	0.063	0.481	0.540	0.045	0.076
Kenpaullone	3820				3.720							0.713				
Keratinocyte Differentiation Inducer	11453158		>25	>25						>25	>25		>25	>25		
Lck Inhibitor	6603792						>25						0.007	0.005		
MEK Inhibitor II	389898	2.191														
Met Kinase Inhibitor	9549297						3.573									
MK-2 Inhibitor III	57269231					3.016										
Olomoucine II	5494414		1.023	0.591												

PDGF Receptor Tyrosine Kinase Inhibitor II	5330548				>25										>25	>25
PDGF RTK Inhibitor	16760609												0.057	0.058		
PDK1/Akt/Flt Dual Pathway Inhibitor	5113385	0.025			0.088											
PI 3-Kg Inhibitor	5289247					2.275										
PI 3-Kα Inhibitor VIII	81055084	0.308	0.168	0.278		0.274	0.202	0.481	2.931	0.441	0.529	0.841	0.586	0.691	0.841	0.492
PI 3-Kγ Inhibitor VII	233033					1.600										
PI 3-Kγ/CKII Inhibitor	26759376					0.192										
PIM1 Kinase Inhibitor II	1235170				9.422					>25	>25					
PIM1/2 Kinase Inhibitor V	2864586					3.809										
PKCb Inhibitor	6419755														2.135	2.852
PKR Inhibitor	6490494		0.094	0.135	3.546	2.387		1.514	13.259						0.071	0.053
PKR Inhibitor, Negative Control	16760619					1.918									1.055	0.792
Purvalanol A	4987		0.503	0.204			1.230	2.945	16.676	0.368	0.738	0.907	0.220	0.243		
Quercetagetin	5281680	0.589			5.214	2.340				2.089	>25		3.319	3.262		
Reversine	210332				5.776		0.170	0.496	2.224	0.441	0.425	0.612	0.485	0.549	1.814	1.353
Rho Kinase Inhibitor V	25093233	2.681														
Ro-31-8220	5083	0.084	0.147	0.318		0.070									0.552	0.503
Roscovitine, (S)-Isomer	6603989		3.577	1.171												
SB 218078	3387354	0.352	0.283	0.871		0.001	0.158	0.438	13.661			0.105	3.611	6.606		
Scytonemin, *Lyngbya* sp.	5486761	8.215						11.843	>25							
Src Kinase Inhibitor I	1474853												0.129	0.374		
Staurosporine, N-benzoyl-	16760627	0.526					0.686	0.219	14.954				0.179	0.225		
Staurosporine, Streptomyces sp.	451705	0.040	0.729	2.514	0.073	0.059	0.004	0.178	1.506	0.003	0.005	0.005	0.172	0.178	0.008	0.052
Ste11 MAPKKK Activation Inhibitor	1474860				4.707								0.663	0.913		
SU11652	5329103	11.705			0.178		0.482	0.174	1.953	0.317	0.395	0.011	0.973	1.260	0.236	0.236

SU9516	5289419		0.725	0.448											0.537	0.294
Syk Inhibitor	6419747	7.807					1.374	0.787	13.500				2.043	2.809		
Syk Inhibitor II	16760670						0.463	0.993	14.827	0.919	0.723	0.590			7.174	7.491
Tpl2 Kinase Inhibitor	9549300	14.246														
TX-1918	6419746	17.703														
UCN-01	72271	0.144	1.431	1.565		0.376	0.134	0.361	1.771	0.018	0.029	0.085				
VEGF Receptor 2 Kinase Inhibitor I	6419834									>25	>25					
VEGF Receptor 2 Kinase Inhibitor III	5329098				0.829		1.422									
Wee1 Inhibitor	10384072	6.506	10.863	4.508		0.239	0.857	0.878	3.331	0.898	0.688	1.554				
Wee1 Inhibitor II	10319891					0.235				>25	>25				2.701	1.564
Wee1/Chk1 Inhibitor	16760707				0.303	0.277						3.675				
WHI-P180, Hydrochloride	5687												1.185	1.602		
